# Long Covid Symptom Clusters, Correlates and Predictors in a Highly Vaccinated Australian Population in 2023

**DOI:** 10.1111/hex.70273

**Published:** 2025-05-08

**Authors:** Essa Tawfiq, Rosalie Chen, Damian Alexander Honeyman, Rebecca Dawson, Mohana Kunasekaran, Adriana Notaras, Deepti Gurdasani, Helen Skouteris, Darshini Ayton, Chandini Raina MacIntyre

**Affiliations:** ^1^ Biosecurity Program The Kirby Institute, Faculty of Medicine and Health The University of New South Wales Sydney Australia; ^2^ University of Western Australia Medical School University of Western Australia Perth Australia; ^3^ William Harvey Research Institute Queen Mary University of London London UK; ^4^ Health and Social Care Unit School of Public Health and Preventive Medicine, Monash University in collaboration with Monash Health Melbourne Australia; ^5^ College of Public Service & Community Solutions, and College of Health Solutions Arizona State University Tempe Arizona USA

**Keywords:** Australia, Covid‐19, long Covid, Omicron, SARS‐CoV‐2

## Abstract

**Background:**

Limited data exists regarding long Covid burden following Omicron infection in highly vaccinated populations.

**Objective:**

To (1) characterise long Covid prevalence and predictors and (2) identify key symptom clusters and their correlates among long Covid patients, during an Omicron‐predominant period in a highly vaccinated population.

**Design:**

Anonymous, online, cross‐sectional survey.

**Setting:**

January 2023, Australia.

**Participants:**

Residents aged ≥ 18 years with self‐reported history of test‐positive Covid‐19.

The main variables studied were socio‐demographic characteristics, Covid‐19 risk factors, vaccination, infection history and experiences with long Covid.

**Main Outcome Measures:**

Long Covid symptoms. Symptom‐based clustering was used to identify long Covid symptom clusters and their functional correlates. Predictors of long Covid occurrence and severity were assessed using multivariable logistic regression.

**Results:**

Overall, 240/1205 participants (19.9%) reported long Covid. Long Covid risk was significantly higher for women OR 1.71 (95% CI: 1.17–2.51), people with comorbidities 2.19 (95% CI: 1.56–3.08) and those using steroid inhalers for Covid‐19 treatment (2.34 [95% CI: 1.29–4.24]). Long‐Covid risk increased with increasing Covid‐19 infection severity (moderately severe symptoms: 2.23 [95% CI: 1.50–3.30], extremely severe symptoms: 5.80 [95% CI: 3.48–9.66], presented to ED/hospitalised: 7.22 [95% CI: 3.06–17.03]). We found no significant difference in the likelihood of long Covid between the Omicron and pre‐Omicron periods, vaccination status and participant age.

We identified two long Covid clusters (pauci‐symptomatic, *n* = 170, vs. polysymptomatic, *n* = 66). Polysymptomatic cluster membership was associated with worse functioning (impacts on work, moderate activity, emotions and energy). Severity acute infection was strongly predictive of polysymptomatic cluster membership (5.72 [2.04–17.58]). Monoclonal antibody treatment was strongly associated with pauci‐symptomatic cluster membership (0.02 [0.00–0.13]).

**Discussion:**

Our study shows that long Covid is an important health burden in Australia, including during the Omicron era, and identifies several risk factors. We found a subgroup of patients characterised by more symptoms and worse functional outcomes. Our findings can inform policies for protecting vulnerable populations and frameworks for long Covid risk assessment and management.

**Conclusions:**

One‐in‐five people may suffer long Covid after acute Covid‐19 infection, with similar risk across age groups. Omicron variants appear not to have a lower risk compared to earlier variants in our study. A cumulative number of symptoms can help triage long Covid patients.

**Patient or Public Contribution:**

We did not involve patients or the public in the design of the questionnaire. However, after a soft launch, we revised some survey questions by reviewing early responses from patients and the public.

## 1 Introduction

Australia experienced one of the largest SARS‐CoV‐2 epidemics in the Asia‐Pacific region between November 2021 and January 2023, largely caused by Omicron [1]. Symptoms that continue after acute SARS‐CoV‐2 infection are referred to as ‘long‐COVID’, defined by the World Health Organization (WHO) as the continuation or development of new symptoms 3 months after SARS‐CoV‐2 infection, lasting at least 2 months without other explanation [2]. However, the lack of a global consensus definition and the differences in study designs, including measured symptoms, context (community/hospital), age distribution and vaccination coverage [3], have complicated the estimation of long Covid prevalence. Wide‐ranging estimates between 4.7% and 80% have been published between 2021 and 2022 [4], while more recent studies have reported point prevalence estimates of 6%–7% in adults and ~1% in children [5, 6, 7, 8, 9]. The global estimated cumulative incidence of long Covid by the end of 2023 was approximately 400 million [10].

Long Covid is a heterogeneous condition with a broad range of symptoms and multisystemic pathology [10, 11]. Over 100 long Covid symptoms have been described [12]. A recent systematic review and meta‐analyses found that fatigue, disturbed sleep and breathlessness were highly prevalent among hospitalised, non‐hospitalised and mixed cohorts [13]. Clinical diagnosis and management are challenging, due to the variable symptom profile, potentially lengthy symptom duration and lack of diagnostic and referral protocols [10, 14]. Factors, such as hospitalisation, severe acute illness, female gender, older age, lower socioeconomic status and the presence of comorbidities, have been associated with long Covid [15, 16, 17, 18, 19, 20]. Whilst symptomatic infection is more likely to cause long Covid, so can asymptomatic infection [21, 22]. Given the association between severe acute illness and long Covid, treatments that reduce the severity of acute Covid‐19 infection, such as antivirals, inhaled corticosteroids and monoclonal antibodies [23, 24, 25], may modulate long‐Covid risk, with one systematic review reporting a protective effect with antivirals, but not monoclonal antibodies [26].

A recent Australian survey found 18.2% of respondents from a highly vaccinated population had ongoing symptoms 90 days after a Covid‐19 infection; however, this study was restricted to Western Australian residents [27]. Reduced clinical severity and risk of hospitalisation have been assumed for the Omicron variant compared to the Delta variant, although much of the data are confounded by vaccination rates, which were higher globally by the end of 2021 when Omicron emerged [28]. Some studies suggest Omicron is less likely to cause long Covid compared to prior variants [29, 30]. The Australian context presents a unique opportunity to characterise long Covid during the Omicron era, as there was relatively low community SARS‐CoV‐2 transmission before Omicron's emergence, due to border closures and other public health orders [31]. Two‐dose vaccination rates in Australians aged 16 years and over were 95% by the end of 2021, when the Omicron wave commenced [32]. We therefore aimed to identify the prevalence of long Covid during an Omicron‐dominant period in a highly vaccinated population in Australia and assess the predictors of long‐Covid risk. We also aimed to assess if any long Covid symptom‐based patient subgroups appeared in the data and to characterise them by phenotypic clusters and functional outcomes.

## 2 Methods

### 2.1 Study Design and Participants

**Figure 1:**
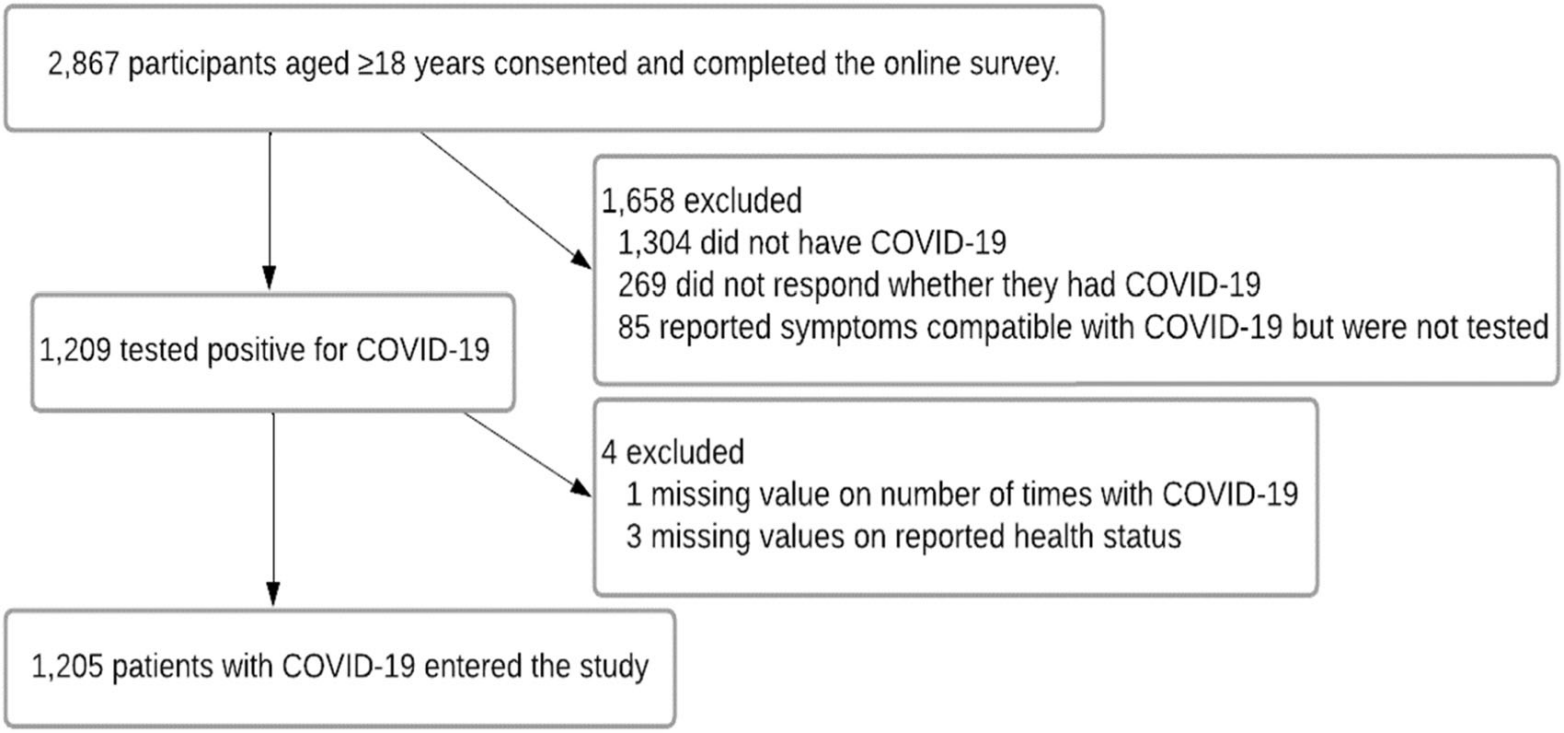
Study population and inclusion and exclusion criteria.

An anonymous, online, cross‐sectional survey of Australian residents aged ≥ 18 years was conducted between 9 and 25 January 2023. Eligible subjects were initially identified using a global market research company (Dynata) [33]. Data on socio‐demographic characteristics, Covid‐19 risk factors, pre‐existing health status, vaccination and infection history were collected using the web‐based survey platform Qualtrics (for further details, see Supporting Information, Methods—Additional Information). Participants were included in this study if they reported testing positive for SARS‐CoV‐2 using a polymerase chain reaction (PCR) test and/or rapid antigen test (RAT) (Figure 1).

### 2.2 Outcomes

The outcome of ‘long COVID’ was based on the WHO Delphi definition [2]. Respondents were asked whether they had ‘symptoms that occurred *within* 3 months of the onset of Covid‐19 and lasted for at least 2 months’. This differs from the WHO definition in that, theoretically, this may include participants who had continuous symptoms for 2 months post‐Covid that resolved within 3 months (i.e., participants who did not have symptoms at 3 months). For those who answered ‘yes’, data on the type and duration of a list of pre‐specified long‐Covid symptoms was collected, with a textbox field for other symptoms. We also asked about changes in self‐reported health status (Excellent, Very good, Good, Fair and Poor) pre‐ and post‐acute Covid‐19 infection based on participant recall at the time of survey. The percentage change in health rating was calculated as the difference in number of patients in each rating category pre‐ and post‐Covid‐19, divided by the number of patients in the pre‐Covid‐19 rating category.

### 2.3 Predictors

We examined a pre‐specified list of predictors based on evidence from the literature from studies examining predictors for long Covid and/or acute Covid‐19 severity (as many studies have shown that acute Covid‐19 severity correlates with risk of long Covid) [14, 34] (Figure S1). We considered age, gender, socio‐economic status, pre‐existing health status, comorbidities, dominant variant at the time of first infection, severity of acute infection, number of infections and vaccination as predictors, given these had been associated with long Covid risk in previous studies [35, 36, 37, 38, 39]. We also included treatments (oral antivirals, monoclonal antibodies, steroid inhalers and other medications) for acute‐Covid‐19, as acute Covid‐19 severity is known to be associated with long Covid [40]. We also examined the association of mask use with long Covid, given viral dosage has been hypothesised to be associated with acute Covid‐19 severity (Figure S1) [41]. We used employment status, house ownership, whether the participant was born in Australia, education level, type of accommodation and family size as possible socio‐economic indicators (see Supporting Information for details). We note that all exposures collected through the questionnaire were based on participant recall at the point of survey, and characteristics such as employment would reflect participant status at the point of survey rather than infection. For information on re‐infection, we only collected the total number of infections at the point of survey, which may not reflect the number of infections before the development of long Covid, leading to misclassification, and conservative estimates of results. We classified the variant causing infection based on the first infection, which may or may not have been the cause of long Covid, which may again lead to misclassification, and conservative estimates.

#### 2.3.1 Statistical Analysis

We first conducted a descriptive analysis of the baseline characteristics of participants who reported infection, with and without long Covid. We used the *χ* ^2^ test to evaluate the statistical significance of differences in baseline characteristics (excluding cells with counts of < 5) (Supporting Information, Methods—Additional Information). To examine the change in health following the development of long Covid, we first calculated the proportion of participants who had worsening health, or improving health, or whose health was stable, post‐long Covid. We then carried out a one‐sample test for difference in proportion from 0 for the proportion with worsening health. This assesses whether the proportion of those with worsening health was statistically significantly different from zero.

To examine predictors of long COVID, we used multivariate logistic regression. We first examined the pre‐specified list of predictors based on the literature (Figure S1), as outlined above (Model 1). To avoid multicollinearity and overfitting, we also carried out backwards selection, iteratively removing the variable with the highest *p* value, until all variables included had a *p* value of < 0.10, and presented these in a second model (Model 2).

Variables included in the full model were age (continuous), gender (male/female), employment status at the time of survey (employed/unemployed), home ownership (yes/no), being born in Australia (yes/no), education level (primary school, high school, TAFE or higher education), type of accommodation (house vs. unit/townhouse/other), self‐reported health status before Covid‐19 infection (poor vs. good), mask use (yes/no), vaccination status (0–2 doses vs. ≥ 3 doses), presence of comorbidities (at least one comorbidity vs. no comorbidity), infection with pre‐Omicron versus Omicron variant (first positive Covid‐19 test before December 2021 vs. from December 2021 onwards) [30], number of Covid‐19 infections (ordinal), severity of acute Covid‐19 infection (mild, moderately severe symptoms, extremely severe symptoms and presented to emergency department) (Table S1), and Covid‐19 treatment with antivirals (yes/no), steroid inhalers (yes/no), monoclonal antibodies (yes/no) and other drugs taken for Covid‐19 treatment (no treatment with these drugs/treated with hydroxychloroquine, ivermectin, fluvoxamine or fluoxetine). When assessing steroid inhalers as a treatment for Covid‐19, patients with pre‐existing respiratory illness (asthma, chronic bronchitis or COPD) were not considered as having received this specifically as treatment for acute COVID, as we were interested in evaluating these as a treatment specifically for acute Covid‐19 infection. We note that considering this group as not having received these specifically for long Covid may lead to more conservative results, biasing our effect estimates towards the null, as some in this group may have received these specifically for acute infection. Categories with small cell counts were excluded from regression analysis (see Supporting Information, Methods—Additional Information).

We examined the assumption of linearity on the logit scale of continuous or ordinal variables using the Box–Tidwell test [42] and found that this was violated for the number of pre‐existing comorbidities when coded as an ordinal variable. This was not violated for any of the other continuous or ordinal variables. We recoded pre‐existing comorbidities as a binary category, as detailed above, with no comorbidity as the reference. Given the multiple predictors tested for, we used a Bonferroni‐corrected threshold for *α*. To maintain an *α* of 0.05, we used a two‐way *p* value threshold of < 0.003 for statistical significance (corrected for 18 tests). We note that, given the conservative nature of Bonferroni correction, our results are likely to be conservative, strictly controlling the false discovery rate at the cost of reduced statistical power. To provide a less conservative comparator, we also present the Benjamini–Hochberg correction [43] using FDR of 5% to calculate adjusted Benjamini–Hochberg *p* values based on ranked *p* values for 18 predictors of long Covid. Those below 0.05 were considered statistically significant in this exploratory analysis.

We also conducted sensitivity analyses to examine the effect of re‐categorising variables differently: vaccination status (unvaccinated, 1–2 doses and ≥ 3 doses), number of COVID‐19 infections (once, twice and three or more times) and age as a categorical variable (18–50, 51–64 and ≥ 65 years).

Given that the severity of acute Covid‐19 and the presence of comorbidities are strongly associated both with long Covid severity (Figure S1), and treatment eligibility, we assessed the potential for residual confounding of the association between treatments and long Covid by these variables. To better assess potential residual confounding of the association between treatments and long Covid by acute Covid severity, and pre‐existing co‐morbidities, we first examined the distribution of Covid‐19 treatments across strata of acute Covid‐19 severity and comorbidities. We then conducted subgroup analyses, examining the relationship between Covid‐19 treatments and long Covid within strata of acute Covid‐19 severity (mild, moderately severe, and extremely severe or presented to ED/hospitalised) and comorbidities (no comorbidity, 1 comorbidity and ≥ 2 comorbidities).

We also examined whether symptoms of long Covid varied between the participants who reported first infections during the pre‐omicron and omicron period. We used the *χ* ^2^ test to examine differences in cell counts for those with and without each symptom.

Our sample of 1205 participants with reported SARS‐CoV‐2 infection allows estimation of long Covid prevalence at a true prevalence of 10% with 2% precision. Our sample of 240 and 965 participants, with and without long Covid, respectively, provides 80% power to detect a proportional difference of 3.4% for exposures present at 20% prevalence in the non‐long‐Covid group with an *α* error of 0.05.

### 2.4 Clustering Analysis

To identify subgroups of participants by symptoms, we carried out clustering analysis across all symptoms, excluding those with low counts ( < 5 participants). As different methods of clustering can result in different subgroups, and there is no single method that is the gold standard, we first conducted an exploratory analysis of two clustering algorithms, Partitioning Around Medoids (PAM) and Agglomerative Hierarchical clustering, both widely used approaches for unsupervised clustering of clinical data [44]. Clustering was performed in ‘R’ (version 2023.03.1+446), using the ‘cluster’ and ‘stats’ packages [45, 46]. Participants were clustered on 19 unique long Covid symptoms recorded, each coded as a binary variable. A Manhattan distance matrix was used to quantify dissimilarity between participants based on symptoms and assign them to clusters. We note that for binary data, the Manhattan distance is equivalent to Hamming distance, an effective measure for comparing two equal‐length binary strings [47].

Average Silhouette coefficient score, a measure of intra‐cluster cohesion and inter‐cluster separation [48] with a range from −1 to +1, was calculated to identify the optimal number of clusters for both methods. A greater positive score indicates that a datapoint is similar to datapoints in the same cluster and different to datapoints in another cluster; a greater negative score indicates likely misclassification. Cluster stability for each method was assessed with Jaccard's coefficient using bootstrapping with 100 resamples to evaluate the extent to which random resampling altered cluster membership. We also assessed the similarity of clusters between the two methods with Jaccard's coefficient. This compares the similarity of clusters identified by calculating a ratio of their intersection to the union size. A value of 0 indicates completely different sets, and 1 indicates completely identical sets.

Participant characteristics, including demographic and socio‐economic variables, vaccination status, severity of Covid‐19 infection and treatment types, were summarised for each cluster, with statistical differences evaluated using Pearson's *χ* ^2^ test for categorical data (excluding cell counts < 5) and Kruskal–Wallis test for ordinal and continuous data.

To examine the functional and clinical significance of symptom clusters, we also examined functional correlates of cluster membership, including physical limitation, emotional status, social impacts, occupational consequences and self‐ratings of health post‐COVID. Pearson's *χ* ^2^ test for categorical data (excluding cell counts < 5) and Kruskal–Wallis test for ordinal and continuous data were used to compare baseline characteristics and functional correlates between clusters. A multiple logistic regression model was fit using the same predictors as used in the multivariate model for long Covid risk, using cluster membership as the dependent variable, to identify predictors of cluster membership. Consistent with our statistical analysis of predictors of long Covid, we present two models for the prediction of cluster membership, one with all pre‐specified potential predictors included, and one using backwards regression, using a threshold of *p* < 0.10. Results are presented in tables, butterfly charts, forest and alluvial plots, created using R (‘plotrix’, ‘metafor’ and ‘ggalluvial’ packages) and MATLAB [49].

### 2.5 Ethical Considerations

This study was approved by the UNSW Human Research Ethics Committee (approval number HC220737).

## 3 Results

One in five (240/1205 [19.9%]) participants in our study reported long Covid symptoms. Statistically significant differences in baseline characteristics (Table 1) were observed between those with and without self‐reported long Covid, with long‐Covid patients being younger adults of working age (*χ* ^2^ = 13.98, *p* = 0.001), disproportionately female (*χ* ^2^ = 17.60, *p* = 0.001) and more likely to report reinfection (*χ* ^2^ = 20.65, *p* < 0.001) and pre‐existing comorbidities (*χ* ^2^ = 18.17, *p* < 0.001). However, there was no statistically significant difference by vaccination status and whether first infection occurred during or before the Omicron‐dominant era (Table 1). Consistent with pre‐existing conditions, and severe acute COVID‐19 being overrepresented among those with self‐reported long COVID, this group were also more likely to report molnupiravir (*χ* ^2^ = 11.39, *p* = 0.001), steroid inhaler (*χ* ^2^ = 32.13, *p* < 0.001) and monoclonal antibody (*χ* ^2^ = 50.56, *p* < 0.001) use compared to those without long Covid.

**Table 1:** Characteristics of participants who had Covid‐19 at time of survey.

		Patients with long Covid	Patients without long Covid	Total number of patients	*p* value
		*n* = 240 (19.9%)	*n* = 965 (80.1%)	*n* = 1205 (100%)	
Age					0.001
18–50 years		179 (22.7%)	611 (77.3%)	790 (100%)	
51–64 years		40 (17.9%)	183 (82.1%)	223 (100%)	
≥ 65 years		21 (10.9%)	171 (89.1%)	192 (100%)	
Gender					0.001
Male		65 (15.0%)	367 (85.0%)	432 (100%)	
Female		170 (22.3%)	594 (77.7%)	764 (100%)	
Non‐binary		< 5	< 5	6 (100%)	
Did not state		< 5	< 5	< 5	
Employment					0.006
Employed		178 (22.1%)	626 (77.9%)	804 (100%)	
Unemployed		62 (15.5%)	339 (84.5%)	401 (100%)	
State/Territory					0.20
Australian Capital Territory		38 (20.7%)	146 (79.3%)	184 (100%)	
New South Wales		30 (20.5%)	116 (79.5%)	146 (100%)	
Northern Territory		15 (22.7%)	51 (77.3%)	66 (100%)	
Queensland		29 (18.2%)	130 (81.8%)	159 (100%)	
South Australia		28 (17.3%)	134 (82.7%)	162 (100%)	
Tasmania		28 (23.7%)	90 (76.3%)	118 (100%)	
Victoria		35 (20.2%)	138 (79.8%)	173 (100%)	
Western Australia		37 (18.8%)	160 (81.2%)	197 (100%)	
Vaccination status					0.19
Unvaccinated		9 (14.1%)	55 (85.9%)	64 (100%)	
1–2 dose(s)		58 (24.2%)	182 (75.8%)	240 (100%)	
3+ doses		172 (19.3%)	720 (80.7%)	892 (100%)	
Unsure whether received vaccines		1 (11.1%)	8 (88.9%)	9 (100%)	
Pre‐existing comorbidity*					< 0.001
Yes		168 (24.1%)	529 (75.9%)	697 (100%)	
No		72 (14.2%)	436 (85.8%)	508 (100%)	
Omicron as the cause of illness					0.38
Yes		188 (19.4%)	780 (80.6%)	968 (100%)	
No		52 (21.9%)	185 (78.1%)	237 (100%)	
Number of times with Covid‐19					< 0.001
1 time		180 (17.9%)	828 (82.1%)	1008 (100%)	
2 times		45 (27.8%)	117 (72.2%)	162 (100%)	
≥ 3 times		15 (42.9%)	20 (57.1%)	35 (100%)	
Severity of acute Covid‐19					< 0.001
Mild		39 (9.2%)	385 (90.8%)	424 (100%)	
Moderately severe		131 (20.9%)	497 (79.1%)	628 (100%)	
Extremely severe		52 (42.6%)	70 (57.4%)	122 (100%)	
Presented to ED/hospitalised		18 (58.1%)	13 (41.9%)	31 (100%)	
Antiviral medications					
Molnupiravir used	Yes	19 (38.8%)	30 (61.2%)	49 (100%)	0.001
	No	221 (19.1%)	935 (80.9%)	1156 (100%)	
Paxlovid used	Yes	22 (28.2%)	56 (71.8%)	78 (100%)	0.06
	No	218 (19.3%)	909 (80.7%)	1127 (100%)	
Remdesivir used	Yes	7 (29.2%)	17 (70.8%)	24 (100%)	0.25
	No	233 (19.7%)	948 (80.3%)	1181 (100%)	
Steroid inhalers for Covid‐19 treatment					< 0.001
Yes		32 (46.4%)	37 (53.6%)	69 (100%)	
No		208 (18.3%)	928 (81.7%)	1136 (100%)	
Monoclonal antibodies for Covid‐19 treatment					< 0.001
Yes		37 (52.9%)	33 (47.1%)	70 (100%)	
No		203 (17.9%)	932 (82.1%)	1135 (100%)	

*Note:* The Bonferroni corrected *p* value was 0.003, based on a 0.05 *α* level and 18 predictors.

* See Figure S2 for a detailed breakdown of comorbidities.

Of the 240 participants with long Covid, a decline in health status was seen post‐Covid (Figure S3 and Table S2), with a statistically significant proportion (52%) reporting a decline in health (*p* value < 0.0001), only 1% reporting an improvement and 47% stable health post‐Covid (Figure S3). Tiredness was the most reported long Covid symptom (18.5%) followed by shortness of breath (12.0%) (Figure S4).

**Figure 2:**
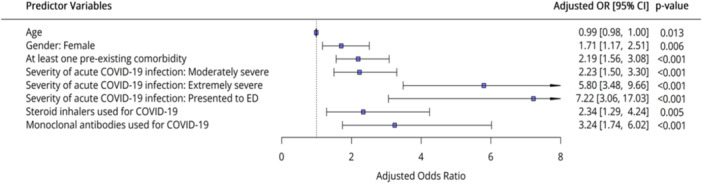
Predictors of long Covid—multivariable regression model (Model 2).

On multivariate regression (Figure 2 and Tables S3 and S4), severe acute Covid‐19 infection was a strong predictor of long Covid, with double the risk (2.23 [95% CI: 1.50–3.30], *p* = 0.0001) for moderately severe symptoms, nearly six times for extremely severe symptoms (5.80 [95% CI: 3.48–9.66], *p* = 0.0001), and seven times for presenting to ED (7.22 [95% CI: 3.06–17.03], *p* = 0.0001), compared to mild illness. Having at least one pre‐existing comorbidity was associated with twice the odds of long Covid (2.19 [95% CI: 1.56–3.08], *p* = 0.0001). Steroid inhaler and monoclonal antibody use for the treatment of Covid‐19 were associated with higher risk (2.34 [95% CI: 1.29–4.24], *p* = 0.005 and 3.24 [95% CI: 1.74–6.02], *p* = 0.001, respectively). Age, female gender and steroid inhaler use were nominally associated with higher risk of long Covid. However, these differences were not statistically significant after Bonferroni multiple correction. However, female gender and steroid inhaler use were statistically significant when using the less conservative Benjamini–Hochberg correction (Table S3). Model 1 (the full model with all pre‐specified variables) and Model 2 (limited model following backwards stepwise regression) both showed consistent effect estimates (Tables S3 and S4). Sensitivity analyses with re‐categorised variables (vaccination status, number of times with Covid‐19 infection and age) produced similar results as Model 1 (Tables S5, S6 and S7 *)*.

Exploratory analyses examining the role of confounding between the association between monoclonal antibody use and long Covid risk showed that monoclonal antibody use was disproportionately overrepresented in those with severe symptoms, or emergency department attendance or hospitalisation, compared to those with mild or moderate symptoms (Table S8). Similarly, monoclonal antibody use was also overrepresented among those with one or more comorbidities (Table S9). Sensitivity analyses within strata of severity showed a statistically significant strong positive association between monoclonal antibody use and long Covid within the mild strata of acute Covid‐19 severity (*p* = 0.001) (Table S10). However, within strata of comorbidities, no statistically significant association was observed between monoclonal antibody use and long Covid (Table S11), although the association was nominally significant (not attaining the threshold for Bonferroni correction) among participants with two or more comorbidities.

We found no material difference between long‐Covid symptoms for participants reporting their first infection in the pre‐Omicron versus Omicron era, except for palpitations, which were more common in those reporting infection in the pre‐Omicron period (6.3%) compared to those in the Omicron period (1.7%), where the difference was statistically significant (*p* < 0.003) (Table S12).

### 3.1 Clustering Analysis

Both clustering methods identified two clusters as the optimal number of clusters (Figures S1 and S5), with a Jaccard's coefficient of 0.86 between methods, suggesting high concordance between the two methods (Figure S6). The Jaccard's coefficient for the clusters within each method was 1.00. As the representation of clusters and functional correlates of these clusters was similar across the two methods, we primarily present results from PAM clustering here (Tables S13–S14 and Figure S7). Results from hierarchical clustering were consistent with this and can be found in Supporting Information (Figures S8–S11 and Tables S15–S17).

For PAM clustering, 236 participants with long Covid were included, with 170 participants assigned to one cluster and 66 to another cluster, with an average silhouette width of 0.39 (Figure S5). We designate these clusters broadly as pauci‐symptomatic and polysymptomatic, as the primary difference between them appears to be number of symptoms reported by participants (median [IQR] symptom count: 3 [1–4] vs. 9 [7–11], *H* = 137.13, *p* = 0.001) (Figure S7). Both clusters had a median (IQR) symptom duration of 3 [2–5] months.

**Figure 3:**
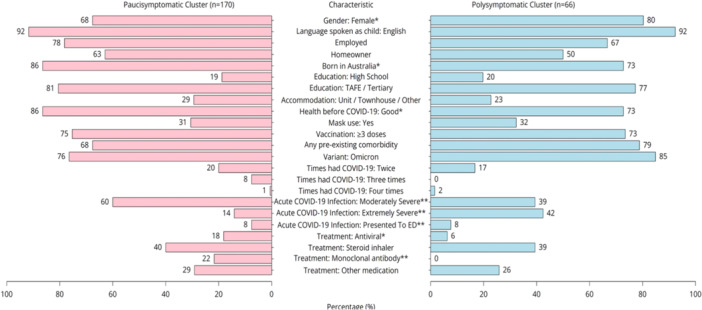
Characteristics of long Covid participants belonging to the pauci‐symptomatic and polysymptomatic clusters at the time of survey. Kruskal–Wallis test was applied to non‐normally distributed continuous variables and ordinal variables. Pearson's *χ* ^2^ was applied to categorical predictor variables. Categories with cell counts less than 5 were excluded from analysis, which included ‘Gender: Non‐binary’. For a detailed frequency table, see Table S13. *p value < 0.05, **p value < 0.001.

On comparing baseline characteristics, the polysymptomatic cluster had a higher proportion of female participants (*χ* ^2^ = 4.91, *p* = 0.03), although this was only nominally significant, and did not attain statistical significance after multiple testing correction. The polysymptomatic cluster also had more participants with severe acute infection (*χ* ^2^ = 22.73, *p* = 0.001) and were less likely to have received monoclonal antibody treatment (*χ* ^2^ = 15.43, *p* = 0.001) (Figure 3).

**Figure 4:**
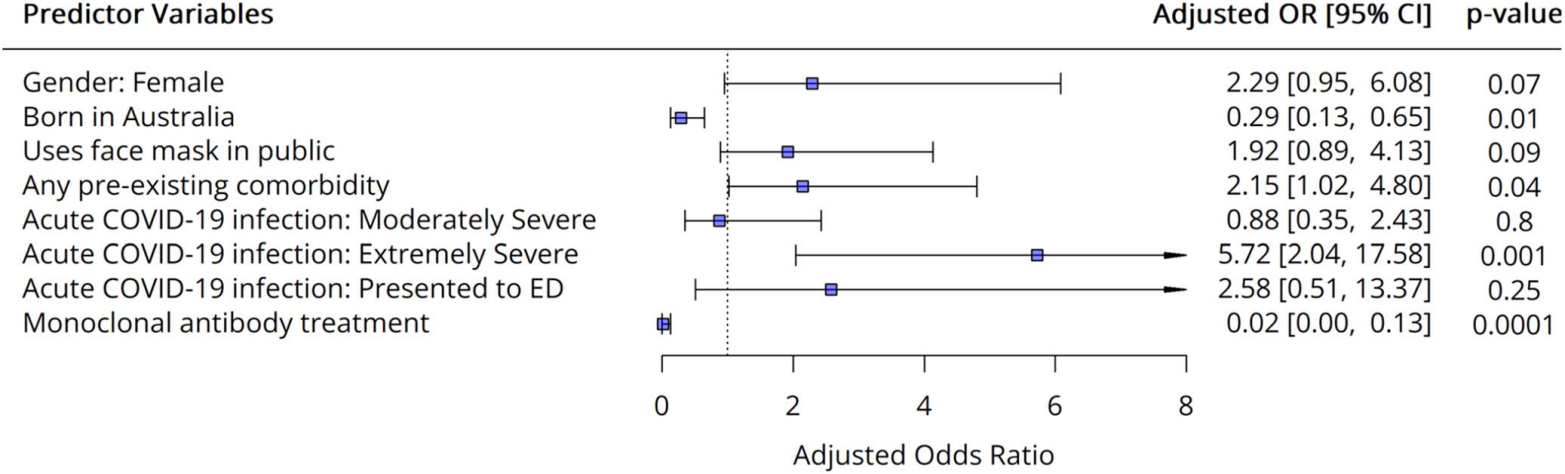
Forest plot showing adjusted odds ratios and 95% confidence interval for predictors of polysymptomatic cluster membership (PAM method). Blue squares indicate the odds ratio, and horizontal lines depict the 95% confidence interval.

In multivariate analysis of predictors of polysymptomatic cluster membership, having Severity of acute infection (extremely severe symptoms in acute infection) was significantly associated with cluster membership (OR = 5.72, 95% CI: 2.04–17.58, *p* = 0.001), and monoclonal antibody treatment was significantly associated with pauci‐symptomatic cluster membership (OR = 0.02, 95% CI: 0.00–0.13, *p* = 0.0001) (Figure 4). Female gender became non‐significant in multivariate analysis.

**Figure 5:**
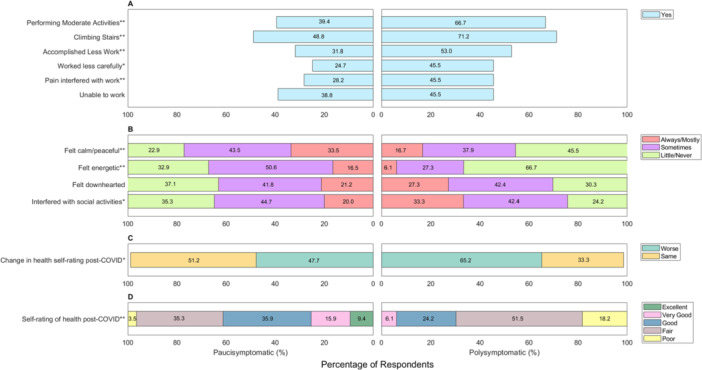
(A–D) Functional correlates and post‐COVID outcomes of long COVID participants belonging to the pauci‐symptomatic and polysymptomatic clusters. Kruskal–Wallis test was applied to non‐normally distributed continuous variables and ordinal variables. Pearson's *χ* ^2^ was applied to categorical predictor variables. Categories with cell counts less than 5 were excluded from analysis, which included ‘Change in health self‐rating post‐COVID: Improved’. For a detailed frequency table, see Table S14. *p value < 0.05, **p value < 0.001.

Regarding functional outcomes, polysymptomatic cluster membership was associated with significantly poorer physical outcomes (limitations with moderate activities [*χ* ^2^ = 14.12, *p* = 0.001] and climbing stairs [*χ* ^2^ = 9.59, *p* = 0.002]), work‐related impacts (more likely to work less carefully [*χ* ^2^ = 9.03, *p* = 0.003], accomplish less work [*χ* ^2^ = 13.0, *p* = 0.001] and report pain interfering with work [*χ* ^2^ = 7.53, *p* = 0.006]) (Figure 5A), emotional status (less likely to feel calm, peaceful [*χ* ^2^ = 12.66, *p* = 0.001] or energetic [*χ* ^2^ = 21, *p* = 0.001] during the last 4 weeks) and social impacts (more likely to report physical or emotional problems interfering with social activities [*χ* ^2^ = 4.99, *p* = 0.03]) (Figure 5B), although this was nominally significant and did not attain statistical significance after multiple testing correction.

Polysymptomatic cluster membership was associated with poor reported health at baseline, with a higher proportion of participants reporting their pre‐infection health status as ‘Poor’ (18/66 [27.27%]), compared to those in the pauci‐symptomatic cluster (23/170 [13.53%]) (Table S13). Those in the polysymptomatic cluster were also more likely to report worsening in health post‐infection compared to baseline (*χ* ^2^ = 5.50, *p* = 0.02) (Figure 5C) and self‐report poorer health status post‐infection (*χ* ^2^ = 25.28, *p* = 0.001) (Figure 5D).

## 4 Discussion

Long Covid was found to impact adults of all age groups in our survey, with a prevalence of nearly 20% in a highly vaccinated population. These findings are consistent with other studies [17, 31], adding to a growing evidence base that suggests the prevalence of long Covid post‐Omicron infection is higher than previously thought and affects all age groups. We found that the Omicron variant was not significantly different to prior variants in causing long Covid, despite previous studies suggesting that the Omicron variant is associated with a decreased long‐Covid risk compared to the Delta variant, even at 6 months post‐vaccination [30]. Although Omicron is believed to be associated with less severe acute infection [29], our findings support evidence that its health impacts may still be substantial, particularly in the form of long Covid, and should not be neglected, even as countries downgrade their severity ratings of SARS‐CoV‐2 infection.

Consistent with previous studies, we also found that female gender, re‐infection, pre‐existing conditions and more severe acute infection were associated with developing long Covid [16, 50, 51]. Women have a fast‐acting, innate and adaptive immune system, which usually protects from initial disease but also increases the risk of chronic immune‐related disorders [52], and they may also have higher exposure to the virus due to caring roles. While abundant research supports that vaccination is associated with reduced long Covid severity and social, personal and work impacts, we did not find an association with vaccination. This may be due to very few participants being unvaccinated, the likely lengthy delays since last booster/vaccination, or residual confounding (e.g., those with pre‐existing conditions are more likely to be vaccinated and to also get long Covid). Most people who reported three vaccination doses would likely have received their last dose at least 6 months before the survey, and a further booster was not yet available at the time of the survey. Immunity to boosters wanes rapidly, even after a third or fourth dose [53]. More research is therefore required to ascertain the relationship between the recency of vaccination and matching of boosters to circulating variants and long‐Covid risk. This is relevant to Australia, where in September 2023, further boosters were only recommended for people aged 75 years and older. For people aged 65–74 years, a booster can be considered 6 months after the last dose or infection, and for people < 65 years, if they are severely immunosuppressed [54]. Our research suggests adults of any age are at risk of long Covid, which should be considered in vaccination policy for all ages.

Antiviral medication has also been found to reduce long Covid risk in unvaccinated and vaccinated cases [55, 56]. However, we found that antivirals were not associated with lower long‐Covid risk, possibly because few participants received antivirals, thus rendering the study underpowered for this predictor, or possibly due to residual confounding, as we discuss subsequently. Monoclonal antibodies were found to be associated with an increased risk of long Covid. This is likely due to residual confounding by the presence of health conditions and acute Covid‐19 severity, which was associated both with antiviral use, monoclonal antibody use and a higher probability of long Covid. Stratifying by the number of comorbidities rendered the association between monoclonal antibodies and long Covid non‐significant (with effect size reduced), although this may also be partly due to reduced statistical power due to smaller numbers within strata.

The exploratory clustering analysis in this study is novel as it assesses phenotypic differences in long Covid patients and their associated baseline characteristics and functional outcomes in a highly vaccinated Australian cohort predominantly infected with Omicron. We identified robust, reproducible and clinically significant clusters of long Covid symptoms. The strong association of the polysymptomatic cluster with more long Covid symptoms and poorer functional outcomes suggests a more severe and multisystemic symptom profile in a smaller subgroup of long Covid patients, consistent with other studies [57]. We show that monoclonal antibody use predicts pauci‐symptomatic cluster membership, suggesting a potentially moderating impact on long Covid symptoms, while more severe acute Covid‐19 disease predicts polysymptomatic cluster membership, which is also associated with poorer physical, emotional, social and occupational impacts. Whichever subgroup one belongs to, therefore, may be determined by a combination of factors, including the severity of acute infection and access to treatment. Understanding these subgroups and their predictors may help clinicians to prioritise patients for diagnostics, specialist referral and more aggressive management.

Our study has several limitations. Our survey may not be generally representative of the Australian population, particularly in terms of geographical location and gender. Most respondents (*n* = 197 [16.3%]) were from Western Australia (the fourth‐most populous state/territory), and New South Wales had 146 respondents (12.1%), despite accounting for 31.4% of Australia's population [58]. Participants were mostly female (*n* = 764 [63.4%]). However, the age distribution and median age of participants (41.0 vs. 38.5 years) and proportion of participants working in healthcare (15.9%) were similar to the general population [58, 59]. Participants were also asked to describe their gender; thus, we could not assess the relationship between sex and long Covid. However, many studies have also reported on the relationship between gender and long Covid [17, 28]. This likely reflects known differences in immunological responses between males and females, with stronger innate immune responses and greater autoimmunity reported in females, influencing the variability in the incidence of autoimmune diseases between sexes [52]. Next, our long Covid definition differs from the Delphi consensus definition, due to the slightly different phrasing of the question, which may lead to inclusion of participants who had continuous symptoms for 2 months post‐Covid that resolved within 3 months (i.e., participants who did not have symptoms at 3 months). While symptoms lasting for 2 months are likely functionally significant to the individual, this may have inflated our estimate of long Covid prevalence relative to the Delphi definition, which requires symptoms to be present at the 3‐month point. Additionally, questions relating to long Covid were asked only of patients who reported testing positive for SARS‐CoV‐2, thus, baseline rates of symptoms could not be determined for those who did not test positive. Non‐response rates also could not be calculated, as Dynata does not provide the total number of survey recipients. Furthermore, recall bias may be present, as patients' SARS‐CoV‐2 infections and symptoms may have happened many months ago. Although we used logistic regression and sensitivity analyses to adjust and assess for confounding, our results could have been affected by residual confounding due to unmeasured confounders, such as time since last Covid‐19 vaccination and acute infection and measurement error in measured confounders. Some data (e.g., median duration of symptoms) were only collected for those who reported having persistent symptoms, so we could not conduct comparisons for such variables between those with and without long Covid. As patient characteristics (e.g., employment) were collected at the point of survey, this may lead to reverse association bias, as characteristics may have been measured following the development of long Covid and may be caused by long Covid, rather than the reverse. We did not collect the dates of each repeat infection, thus, the total number of infections may not indicate the cumulative number of infections before developing long Covid, as long Covid could have developed before the most recent infection. It is also possible that a patient's first and any subsequent infection(s) were caused by different SARS‐CoV‐2 variants. These limitations are expected to have led to misclassification bias, which would have biased results towards the null. There is also a possibility of recall bias for exposures, given that exposures and health status were recalled following infection and/or development of long Covid, which may potentially bias results away from the null. Regarding the clustering analysis based on long Covid symptoms experienced, it is possible that the milder symptom profile in the pauci‐symptomatic cluster may be a result of treatment received, as we found a strong association with monoclonal antibody treatment. We note that while the pauci‐symptomatic cluster is strongly associated with treatment, causality cannot be inferred, given the cross‐sectional and observational nature of our survey. Further research is required to elucidate the effect of multiple infections, possibly due to multiple variants, on long Covid risk and severity, such as investigating whether multiple infections impede recovery from long Covid caused by an earlier infection or if subsequent infections indeed increase long Covid risk. Lastly, as the study is observational, causality between the predictors and the outcome of long Covid cannot be examined.

Overall, our study shows that long Covid is an important health burden in Australia and identifies several potential risk factors for its occurrence and severity. Our findings can inform diagnosis, referral and management of patients based on the cumulative number of symptoms, particularly if combined with better organ system‐based pathological and diagnostic protocols [60]. These findings could also inform policies to protect vulnerable populations, including minimising community transmission, ensuring adequate vaccination coverage and treatment access, particularly for severe acute Covid‐19 infection, producing decision‐support tools for assessing individual long Covid risk and guidelines for long Covid management. Further research is warranted to examine the impact of more recent vaccination, particularly in adults of working age, and multiple SARS‐CoV‐2 infections on long Covid outcomes.

## 5 Conclusion

This study provides further evidence that long Covid presents an important health burden for Australian Covid‐19 survivors of all ages. The Omicron variant appears as likely to cause long Covid as prior variants. We identified several risk factors for long Covid occurrence and severity and found a smaller subgroup of patients who appear to experience a more severe, polysymptomatic profile. Public health policy should support diagnostic, preventive and therapeutic measures for reducing long Covid risk and severity.

## Author Contributions

**Essa Tawfiq:** methodology, writing – original draft, writing – review and editing, formal analysis, investigation, data curation, visualization. **Rosalie Chen:** methodology, investigation, visualization, formal analysis, writing – review and editing, writing – original draft, data curation. **Damian Alexander Honeyman:** writing – original draft, writing – review and editing, methodology, formal analysis, investigation, visualization, data curation. **Rebecca Dawson:** methodology, formal analysis, investigation. **Mohana Kunasekaran:** writing – review and editing, methodology. **Adriana Notaras:** writing – review and editing, methodology. **Deepti Gurdasani:** methodology, supervision, writing – review and editing, data curation, investigation, writing – original draft. **Helen Skouteris:** conceptualization, writing – review and editing. **Darshini Ayton:** conceptualization, writing – review and editing. **Chandini Raina MacIntyre:** conceptualization, methodology, resources, funding acquisition, writing – review and editing, supervision, data curation, investigation, writing – original draft.

## Ethics Statement

This study was approved by the UNSW Human Research Ethics Committee (approval number HC220737).

## Conflicts of Interest

E.T., R.C., D.A.H., R.D., M.K., A.N., H.S. and D.A. authors declare no conflicts of interest. D.G. is a member of OzSAGE. C.R.M. is on the WHO COVID‐19 Vaccine Composition Technical Advisory Group and the WHO SAGE Working Group on Smallpox and Monkeypox. She receives funding from Sanofi for influenza and pertussis research and from NHMRC and MRFF.

## Supporting information

Supporting Materials‐29Mar2025.

## Data Availability

The authors have nothing to report.
