# Supplementary material for: Long Covid Symptom Clusters, Correlates and Predictors in a Highly Vaccinated Australian Population in 2023
Source: Health Expect. 2025 May 8;28(3):e70273. doi: 10.1111/hex.70273 (PMC12059467; doi:10.1111/hex.70273)
Supplement: Supplementary file 1 — Supporting Materials‐29Mar2025. [file HEX-28-e70273-s001.docx]

# **Table of Contents**

[Methods – Additional Information 4](#_Toc185933942)

[The Survey Process 4](#_Toc185933943)

[Figure S1: Directed Graph showing potential relationships between possible causal factors, confounders and long COVID 5](#_Toc185933944)

[Table S1. Definition of acute COVID-19 infection severity based on participant responses to the survey question “How severe was COVID-19 when you had it the first time?” 6](#_Toc185933945)

[Statistical analysis 6](#_Toc185933946)

[Figure S2. Pre-existing comorbidities among participants. 7](#_Toc185933947)

[Figure S3. Health status pre and post-COVID in long-COVID survey participants 8](#_Toc185933948)

[Table S2. Number of participants with long-COVID who reported their health status by severity of acute COVID 8](#_Toc185933949)

[Figure S4. Long-COVID symptoms reported by participant 9](#_Toc185933950)

[Table S3. Full model containing the all pre-specified potential predictors in multivariate analysis of Long-COVID (Model 1) 10](#_Toc185933951)

[Table S4. Predictors of Long-COVID after backward stepwise regression (Model 2) 11](#_Toc185933952)

[Table S5. Sensitivity analysis, using 3 categories of times with COVID-19, applying the full model 12](#_Toc185933953)

[Table S6. Sensitivity analysis, using 3 categories of vaccination status, applying the full model 13](#_Toc185933954)

[Table S7. Sensitivity analysis, using 3 categories of age group, applying the full model 14](#_Toc185933955)

[Table S8. Distribution of data on the use of steroid inhalers, monoclonal antibodies, and oral antivirals among participants with Long COVID and those without Long COVID, by severity of acute COVID-19 15](#_Toc185933956)

[Table S9. Distribution of data on the use of steroid inhalers, monoclonal antibodies, and oral antivirals among participants with Long COVID and those without Long COVID, by status of pre-existing comorbidity 16](#_Toc185933957)

[Table S10. Subgroup analysis by severity of acute COVID-19, using the full model 17](#_Toc185933958)

[Table S11. Subgroup analysis by status of pre-existing comorbidity, using the full model 18](#_Toc185933959)

[Table S12. Long-COVID symptoms by Omicron variant vs. pre-Omicron variants (mainly Delta) 19](#_Toc185933960)

[Clustering Results 20](#_Toc185933961)

[Partitioning Around Medoids (PAM) Clustering Results 20](#_Toc185933962)

[Figure S5. Silhouette plot for two to five clusters using the PAM clustering method. 20](#_Toc185933964)

[Figure S6. Euler plots comparing the pauci-symptomatic and polysymptomatic clusters obtained by PAM and Hierarchical Clustering methods. 21](#_Toc185933965)

[Table S13. Baseline participant characteristics by cluster (Total Participants = 236) using PAM clustering. 22](#_Toc185933966)

[Figure S7. Alluvial plot of severity of acute COVID-19 infection with total number of long-COVID symptoms experienced by participants and long-COVID cluster membership using the PAM clustering method. 24](#_Toc185933967)

[Table S14. Participant functional correlates following COVID-19 infection by cluster membership (Total Participants = 236) by PAM clustering. 25](#_Toc185933968)

[Hierarchical Clustering Results 27](#_Toc185933969)

[Description of Results 27](#_Toc185933970)

[Figure S8. Silhouette plot for 2-5 clusters obtained by hierarchical clustering. 28](#_Toc185933971)

[Figure S9. Scree plot for hierarchical clustering. 29](#_Toc185933972)

[Figure S10. Dendrogram plot depicting clusters obtained using Hierarchical clustering with Manhattan distance and complete linkage. 30](#_Toc185933973)

[Table S15. Baseline participant characteristics by cluster (Total Participants = 236) using hierarchical clustering. 31](#_Toc185933974)

[Table S16. Full model containing all potential predictors of polysymptomatic cluster membership by hierarchical clustering. 33](#_Toc185933975)

[Figure S11. Forest plot showing adjusted odds ratios and 95% confidence interval for predictors of polysymptomatic cluster membership by hierarchical clustering (Model 2). 34](#_Toc185933976)

[Table S17. Participant functional correlates following COVID-19 infection by cluster membership (Total Participants = 236) by hierarchical clustering. 35](#_Toc185933977)

# Methods – Additional Information

## *The Survey Process*

Dynata is a global market research company with experience in conducting medical research population surveys in over 30 countries, including Australia. To recruit participants, Dynata randomly distributed a survey link among a geographically targeted sample of their panel members meeting eligibility criteria from Australia. The link was distributed using email and their online platform. Once participants opened the link, they were redirected to the survey page on Qualtrics. They were asked about their age and state/territory of residence to determine eligibility. If they met the inclusion criteria, they were brought to a landing page with the Participant Information Sheet, which they were asked to read prior to continuing. Participants provided informed consent prior to completing the survey. After proceeding through the Participant Information Sheet, participants were asked to check boxes indicating their consent before proceeding to the online survey. Checking these boxes and completing the survey was considered consent. Because no identifying information was collected, participants were not able to withdraw consent once the survey was completed. Failure to complete the survey in full was considered withdrawal of consent, and any data collected were destroyed. The survey took approximately 15-20 minutes to complete. The survey questions were piloted for time and clarity, then refined and redeployed for the final survey. Two experts from the research team were responsible for managing data collection. A verification process and regular quality checks were used to ensure reliability and accuracy of responses and to avoid duplication of participants. Both had extensive experience in implementing online surveys. Dynata enabled targeted sampling by controlling outbound invitations on pre-determined targeting criteria (age and state/territory of residence). The number of outbound invitations was proportionate to national statistics to improve the representativeness of the sample.

*Predictor Variables*

In light of the literature, we have employed a DAG diagram (below) to assess the relationship between various predictors of long COVID, and between each predictor and long COVID, using a binary logistic regression model.

## **Figure S1: Directed Graph showing potential relationships between possible causal factors, confounders and long COVID**


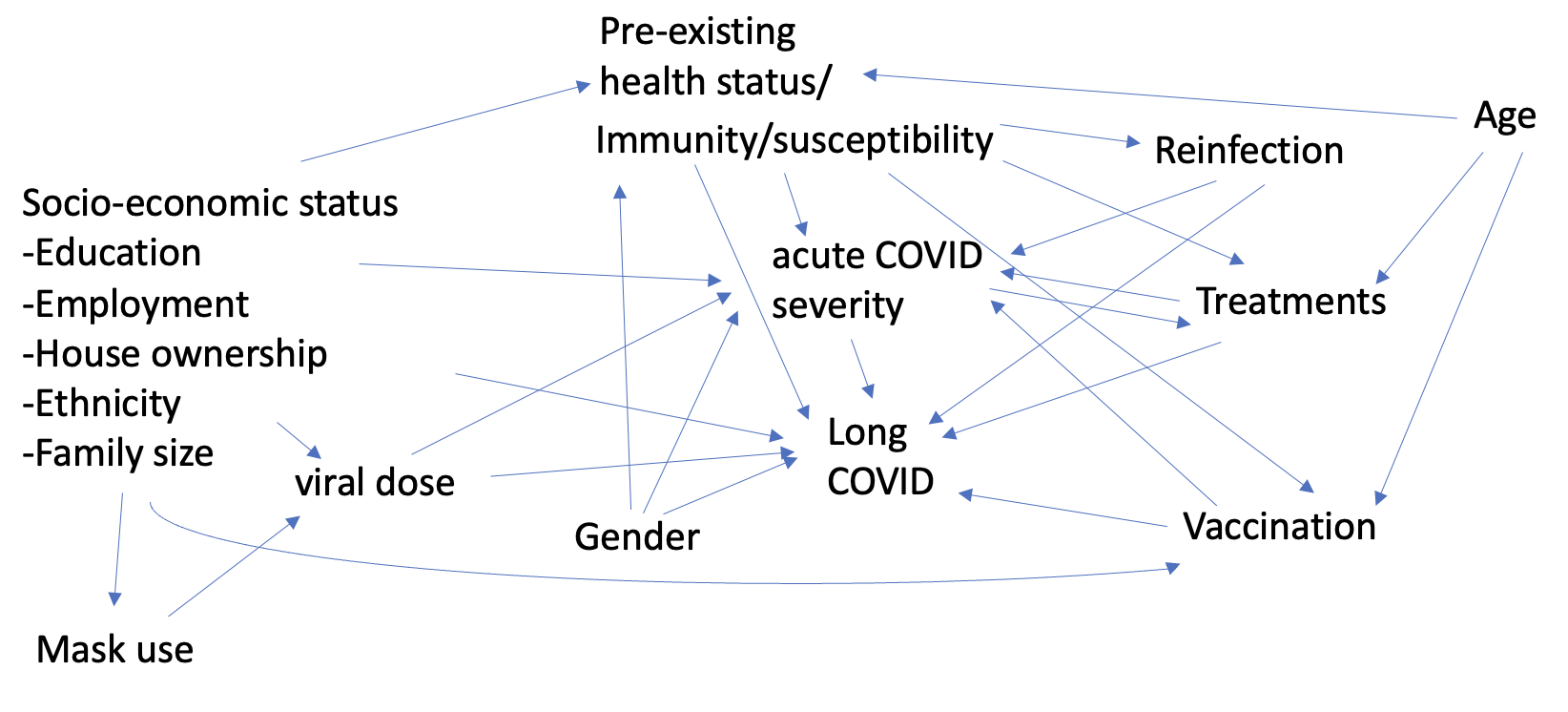


We note that this graph, while directed, is not strictly acyclic as there are bidirectional relationships, for example between acute COVID-19 severity and treatments. For example, severity itself can influence eligibility for treatment, and treatment can influence severity of acute infection. If these causal relationships are considered to occur at different time points, however, this graph can be considered acyclic for a given point in time.

In the multivariable binary logistic regression model, age was included as a continuous variable, gender as a binary variable (female vs. male), home ownership (yes vs. no), being born in Australia (yes vs. no), education level (primary education, high school, TAFE/tertiary), type of accommodation (house vs. townhouse/apartment/other), reported health status (good vs. poor), mask use (yes vs. no), employment status (employed vs. unemployed), number of times had COVID-19 (ordinal variable), infection with pre-Omicron vs. Omicron variant (first positive COVID-19 test before December 2021 vs. from December 2021 onwards), pre-existing comorbidity (no comorbidity vs. ≥1 comorbidities) that ranged from 0 to 11 health conditions that respondents reported from a list of pre-specified illnesses, COVID-19 vaccination status (0-1-2 doses vs. ≥ 3 doses), Severity of acute COVID-19 infection (mild, moderately severe, extremely severe (but not hospitalised), and presented to ED), oral antiviral treatment (yes vs. no as whether the participant received Molnupiravir and/or Paxlovid), use of steroid inhalers for COVID-19 treatment (yes vs. no), and treatment with monoclonal antibodies (yes vs. no), and other drugs taken for COVID-19 (yes vs. no as whether the participant received any of the following: hydroxychloroquine, ivermectin, fluvoxamine, fluoxetine).

As only 64 (5.3% of 1,205) participants were unvaccinated, we did not classify vaccination status as ‘unvaccinated’ vs. ‘vaccinated'. We tested vaccination status in the model as a categorical variable (unvaccinated, 1-2 doses, ≥ 3 doses) and found none of them to be statistically significant. Therefore, a binary variable (≥ 3 doses vs. 0-1-2 doses) was used to examine whether booster vaccine doses could be significant.

For antiviral treatment, 151 subjects received Remdesivir, Paxlovid or Molnupiravir. Those receiving Paxlovid or Molnupiravir were combined for analysis due to small numbers. Patients receiving Remdesivir were considered separately as Remdesivir requires intravenous administration and is given in hospital (29). Participants receiving Remdesivir were ultimately not included in the regression model, due to most responding that they had received the drug outside of hospital.

## **Table S1. Definition of acute COVID-19 infection severity based on participant responses to the survey question “How severe was COVID-19 when you had it the first time?”**

| Severity of acute COVID-19 | Survey Responses |
| --- | --- |
| Mild | Not severe at all |
| Moderately severe | Somewhat severe  Moderately severe |
| Extremely severe | Extremely severe, but did not need to be in hospital |
| Presented to ED | Extremely severe, visited the emergency department  Extremely severe, was admitted to hospital  Extremely severe, was admitted to ICU |

## *Statistical analysis*

The generalized linear model (GLM) for predictors of long-COVID is shown below.

$\boldsymbol{Y}_{\boldsymbol{i}\boldsymbol{j}}=\boldsymbol{\beta}_{\mathbf{0}} +\boldsymbol{\beta}_{\mathbf{1}}\boldsymbol{Omicron}_{\boldsymbol{j}}+\boldsymbol{\beta}_{\mathbf{2}}\boldsymbol{PreexistingComorbidities}_{\boldsymbol{j}}+\boldsymbol{\beta}_{\mathbf{3}}\boldsymbol{SeverityAucteIllness}_{\boldsymbol{j}}+\boldsymbol{\beta}_{\mathbf{4}}\boldsymbol{VaccinationStatus}_{\boldsymbol{j}}+\boldsymbol{\beta}_{\mathbf{5}}{\boldsymbol{Number} \boldsymbol{of} \boldsymbol{times} \boldsymbol{with} \boldsymbol{COVID}\mathbf{19}}_{\boldsymbol{j}}+\boldsymbol{\beta}_{\mathbf{6}}\boldsymbol{AntiViral}_{\boldsymbol{j}}+ \boldsymbol{\beta}_{\mathbf{7}}\boldsymbol{SteroidInhaler}_{\boldsymbol{j}}+ \boldsymbol{\beta}_{\mathbf{8}}\boldsymbol{MonocolonalAntibody}_{\boldsymbol{j}} + \boldsymbol{\beta}_{\mathbf{9}}\boldsymbol{OtherMedications}_{\boldsymbol{j}}+ \boldsymbol{\beta}_{\mathbf{10}}\boldsymbol{HealthStatus}_{\boldsymbol{j}}+ \boldsymbol{\beta}_{\mathbf{11}}{\boldsymbol{Use} \boldsymbol{of} \boldsymbol{mask}}_{\boldsymbol{j}}+Ʃ_{\boldsymbol{k}=\mathbf{1}}^{\boldsymbol{K}}\boldsymbol{\beta}_{\boldsymbol{j}}\boldsymbol{X}_{\boldsymbol{ij}}+ ɛ_{\boldsymbol{ij}}$

$\boldsymbol{Y}_{\boldsymbol{ij}}$ refers to the outcome variable for respondent i (whether she/he had long-COVID), with j predicting variables. $\boldsymbol{\beta}_{\mathbf{1}, \mathbf{2},\mathbf{3},\mathbf{4},\mathbf{5},\mathbf{6},\mathbf{7},\mathbf{8},\mathbf{9},\mathbf{10},\mathbf{11}}$ provides the odds ratio (OR) for Omicron, pre-existing comorbidities, severity of acute COVID-19, vaccination status, number of times with COVID-19, antiviral treatment, steroid inhaler, monoclonal antibody, other medications, health status, and use of mask. $\boldsymbol{X}_{\boldsymbol{ij}}$ denotes a vector of predictors, containing sociodemographic characteristics (i.e., age, gender, education level, house ownership, accommodation type, born in Australia, and employment status), and $\boldsymbol{k}$ refers to the number of predictors. β_0_ stands for the intercept term, and β_j_ refers to the OR for each predictor of sociodemographic characteristics. $ɛ_{\boldsymbol{ij}}$refers to the error term.

# **Figure S2. Pre-existing comorbidities among participants.**


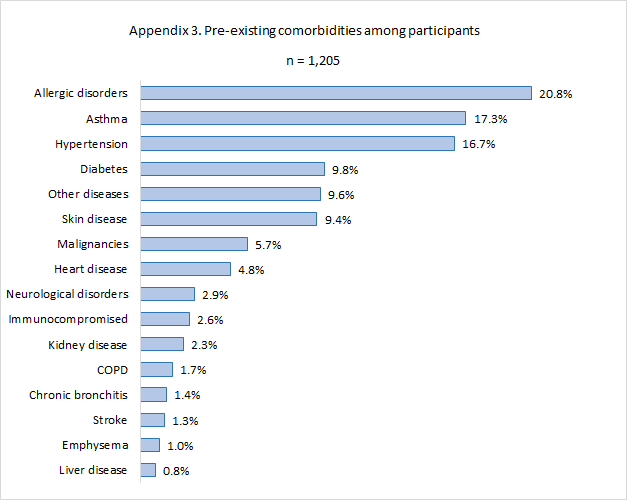


#

# **Figure S3. Health status pre and post-COVID in long-COVID survey participants**


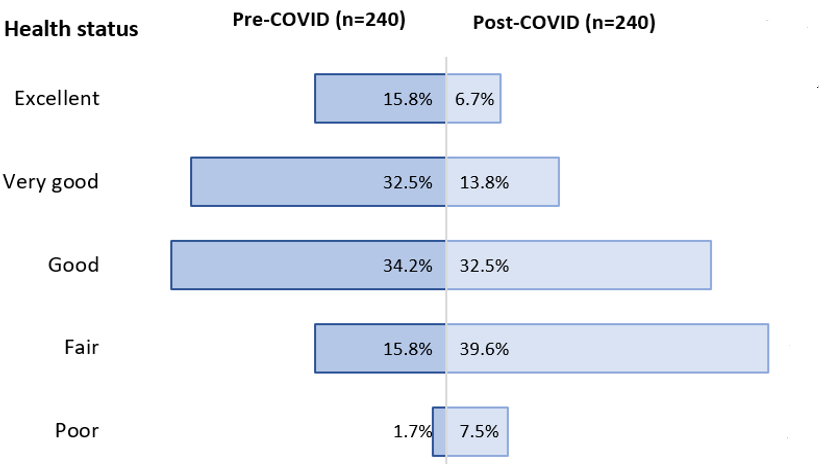


# **Table S2. Number of participants with long-COVID who reported their health status by severity of acute COVID**

| Symptom | Health status | Pre-COVID-19 | Post-COVID-19 |
| --- | --- | --- | --- |
| Mild | Excellent | 7 | 4 |
|  | Very good | 10 | 9 |
|  | Good | 17 | 11 |
|  | Fair | 5 | 13 |
|  | Poor |  | 2 |
| Moderately  severe | Excellent | 16 | 6 |
|  | Very good | 43 | 18 |
|  | Good | 48 | 51 |
|  | Fair | 24 | 47 |
|  | Poor |  | 9 |
| Extremely  severe | Excellent | 9 | 2 |
|  | Very good | 18 | 5 |
|  | Good | 14 | 13 |
|  | Fair | 8 | 28 |
|  | Poor | 3 | 4 |
| Presented to ED | Excellent | 6 | 4 |
|  | Very good | 7 | 1 |
|  | Good | 3 | 3 |
|  | Fair | 1 | 7 |
|  | Poor | 1 | 3 |
| Total |  | 240 | 240 |

# **Figure S4. Long-COVID symptoms reported by participant**

# **Table S3. Full model containing the all pre-specified potential predictors in multivariate analysis of Long-COVID (Model 1)**

| Variable n=1,187 |  | OR (95%CI) | p-value | Benjamini-Hochberg critical p-value |
| --- | --- | --- | --- | --- |
| Age (in years; used a continuous variable) |  | 0.99(0.98-1.00) | 0.16 | 0.279 |
| Female gender | Male (ref) | 1.71(1.16-2.53) | 0.007 | 0.018 |
| Employed | Unemployed (ref) | 1.35(0.90-2.03) | 0.14 | 0.279 |
| Owned house | No (ref) | 0.98(0.69-1.40) | 0.93 | 0.794 |
| Born in Australia | No (ref) | 1.13(0.75-1.70) | 0.55 | 0.620 |
| Education level | Primary school | Ref |  |  |
|  | High school | 0.49(0.11-2.07) | 0.33 | 0.490 |
|  | TAFE/Tertiary | 0.86(0.21-3.57) | 0.83 | 0.789 |
| Type of accommodation | House | Ref |  |  |
|  | Unit/townhouse/other | 0.98(0.69-1.40) | 0.92 | 0.749 |
| Being in good health status | No (ref) | 0.80(0.51-1.24) | 0.31 | 0.512 |
| Agreed to use face mask | No (ref) | 0.92(0.56-1.50) | 0.73 | 0.773 |
| Vaccination status (received ≥ 3 doses) | 0-2 dose(s) (ref) | 0.89(0.60-1.31) | 0.55 | 0.620 |
| At least one comorbidity pre-existed | No (ref) | 2.14(0.150-3.04) | <0.001 | <0.001 |
| Omicron as cause of the illness | No (ref) | 1.43(0.93-2.21) | 0.11 | 0.236 |
| Number of times with COVID-19 (ordinal variable) | 1, 2, 3, 4 time(s) | 1.16(0.84-1.58) | 0.37 | 0.490 |
| Severity of acute COVID-19 infection | Mild | Ref |  |  |
|  | Moderately severe | 2.15(1.44-3.20) | <0.001 | <0.001 |
|  | Extremely severe | 5.96(3.52-10.09) | <0.001 | <0.001 |
|  | Presented to ED | 6.84(2.82-16.60) | <0.001 | <0.001 |
| Oral antivirals used for COVID-19 | No (ref) | 1.19(0.67-2.13) | 0.56 | 0.620 |
| Steroid inhalers used for COVID-19 | No (ref) | 2.43(1.30-4.54) | 0.006 | 0.018 |
| Monoclonal antibodies used for COVID-19 | No (ref) | 3.80(1.70-8.51) | 0.001 | 0.004 |
| Other medications used for COVID-19 | No (ref) | 0.70(0.32-1.55) | 0.38 | 0.490 |

Using p-values of <0.10 from this model, the final model, which contained age, gender, pre-existing comorbidity, severity of acute COVID-19 infection, steroid inhalers and monoclonal antibodies, was obtained for Model2.

# **Table S4. Predictors of Long-COVID after backward stepwise regression (Model 2)**

|  | | | |
| --- | --- | --- | --- |
| Variable n=1,187 |  | OR (95%CI) | p-value |
| Age (continue variable) |  | 0.99(0.98-1.00) | 0.013 |
| Sex (female) | Male (ref) | 1.71(1.17-2.51) | 0.006 |
| At least one comorbidity pre-existed | No (ref) | 2.19(1.56-3.08) | <0.001 |
| Severity of acute COVID-19 infection | Mild (ref) |  |  |
|  | Moderately severe | 2.23(1.50-3.30) | <0.001 |
|  | Extremely severe | 5.80(3.48-9.66) | <0.001 |
|  | Hospitalised | 7.22(3.06-17.03) | <0.001 |
| Steroid inhalers used for COVID-19 | No (ref) | 2.34(1.29-4.24) | 0.005 |
| Monoclonal antibodies used for COVID-19 | No (ref) | 3.24(1.74-6.02) | <0.001 |
| Since in the full model (S12), we tested the model on 18 predictors, here we divided the 0.05 alpha level by 18 and obtained a Bonferroni p-value threshold of 0.003 to interpret results, in light of multiple comparison testing. | | | |

# **Table S5. Sensitivity analysis, using 3 categories of times with COVID-19, applying the full model**

| Variable |  | n=1,187 | p-value |
| --- | --- | --- | --- |
|  |  | adOR (95% CI) |  |
| Age |  | 0.99(0.98-1.00) | 0.15 |
| Female gender | Male (ref) | 1.73(1.17-2.55) | 0.006 |
| Employed | Unemployed (ref) | 1.35(0.90-2.03) | 0.15 |
| Owned house | No (ref) | 0.98(0.69-1.40) | 0.90 |
| Born in Australia | No (ref) | 1.13(0.75-1.70) | 0.56 |
| Education level | Primary (ref) | - | - |
|  | High school | 0.47(0.11-1.99) | 0.31 |
|  | TAFE / Tertiary | 0.83(0.20-3.43) | 0.79 |
| Type of accommodation | House (ref) | - | - |
|  | Unit/townhouse/other | 0.99(0.69-1.41) | 0.94 |
| Being in good health status | No (ref) | 0.80(0.52-1.24) | 0.32 |
| Agreed to use face mask | No (ref) | 0.92(0.56-1.50) | 0.73 |
| Vaccination status | 0-2 doses (ref) | - | - |
|  | ≥3 doses | 0.89(0.60-1.31) | 0.55 |
| At least one comorbidity pre-existed | No (ref) | 2.13(1.50-3.03) | <0.001 |
| Omicron as cause of the illness | No (ref) | 1.41(0.91-2.17) | 0.12 |
| Number of times with COVID-19 | Once (ref) |  |  |
|  | Twice | 1.19(0.76-1.88) | 0.44 |
|  | Three/four | 1.05(0.43-2.57) | 0.92 |
| Severity of acute COVID-19 | Mild (ref) | - | - |
|  | Moderately severe | 2.15(1.44-3.21) | <0.001 |
|  | Extremely severe | 6.02(3.56-10.18) | <0.001 |
|  | Presented to ED | 6.80(2.78-16.59) | <0.001 |
| Oral antivirals used for COVID-19 | No (ref) | 1.19(0.67-2.14) | 0.55 |
| Steroid inhalers used for COVID-19 | No (ref) | 2.42(1.29-4.53) | 0.006 |
| Monoclonal antibodies used for COVID-19 | No (ref) | 4.02(1.79-9.06) | 0.001 |
| Other medications used for COVID-19 | No (ref) | 0.69(0.31-1.53) | 0.36 |

# **Table S6. Sensitivity analysis, using 3 categories of vaccination status, applying the full model**

| Variable |  | n=1,187 | p-value |
| --- | --- | --- | --- |
|  |  | adOR (95% CI) |  |
| Age |  | 0.99(0.98-1.00) | 0.16 |
| Female gender | Male (ref) | 1.71(1.16-2.53) | 0.007 |
| Employed | Unemployed (ref) | 1.33(0.88-1.99) | 0.17 |
| Owned house | No (ref) | 0.99(0.69-1.41) | 0.94 |
| Born in Australia | No (ref) | 1.14(0.76-1.71) | 0.53 |
| Education level | Primary (ref) | - | - |
|  | High school | 0.47(0.11-2.01) | 0.31 |
|  | TAFE / Tertiary | 0.83(0.20-3.46) | 0.80 |
| Type of accommodation | House (ref) | - | - |
|  | Unit/townhouse/other | 0.99(0.69-1.41) | 0.94 |
| Being in good health status | No (ref) | 0.80(0.52-1.24) | 0.32 |
| Agreed to use face mask | No (ref) | 0.89(0.54-1.47) | 0.65 |
| Vaccination status | 0 dose (ref) |  | - |
|  | 1-2 doses | 1.68(0.72-3.94) | 0.23 |
|  | ≥3 doses | 1.37(0.60-3.11) | 0.46 |
| At least one comorbidity pre-existed | No (ref) | 2.16(1.51-3.07) | <0.001 |
| Omicron as cause of the illness | No (ref) | 1.43(0.92-2.20) | 0.11 |
| Number of times with COVID-19 |  | 1.15(0.84-1.58) | 0.37 |
| Severity of acute COVID-19 | Mild (ref) | - | - |
|  | Moderately severe | 2.11(1.42-3.15) | <0.001 |
|  | Extremely severe | 5.88(3.48-9.95) | <0.001 |
|  | Presented to ED | 6.49(2.66-15.87) | <0.001 |
| Oral antivirals used for COVID-19 | No (ref) | 1.18(0.66-2.11) | 0.59 |
| Steroid inhalers used for COVID-19 | No (ref) | 2.38(1.27-4.46) | 0.007 |
| Monoclonal antibodies used for COVID-19 | No (ref) | 4.04(1.79-9.15) | 0.001 |
| Other medications used for COVID-19 | No (ref) | 0.70(0.32-1.56) | 0.39 |

# **Table S7. Sensitivity analysis, using 3 categories of age group, applying the full model**

| Variable |  | n=1,187 | p-value |
| --- | --- | --- | --- |
|  |  | adOR (95% CI) |  |
| Age group | ≥ 65 years (ref) |  | - |
|  | 51-64 years | 1.43(0.73-2.78) | 0.29 |
|  | 18-50 years | 1.54(0.79-3.00) | 0.21 |
| Female gender | Male (ref) | 1.76(1.20-2.58) | 0.004 |
| Employed | Unemployed (ref) | 1.34(0.88-2.03) | 0.17 |
| Owned house | No (ref) | 0.95(0.67-1.34) | 0.75 |
| Born in Australia | No (ref) | 1.14(0.76-1.71) | 0.52 |
| Education level | Primary (ref) | - | - |
|  | High school | 0.50(0.12-2.13) | 0.35 |
|  | TAFE / Tertiary | 0.88(0.21-3.68) | 0.87 |
| Type of accommodation | House (ref) | - | - |
|  | Unit/townhouse/other | 0.98(0.68-1.39) | 0.90 |
| Being in good health status | No (ref) | 0.81(0.52-1.26) | 0.36 |
| Agreed to use face mask | No (ref) | 0.93(0.56-1.52) | 0.76 |
| Vaccination status | 0-2 doses (ref) | - | - |
|  | ≥3 doses | 0.85(0.58-1.25) | 0.41 |
| At least one comorbidity pre-existed | No (ref) | 2.08(1.46-2.94) | <0.001 |
| Omicron as cause of the illness | No (ref) | 1.45(0.94-2.24) | 0.09 |
| Number of times with COVID-19 |  | 1.17(0.85-1.60) | 0.33 |
| Severity of acute COVID-19 | Mild (ref) | - | - |
|  | Moderately severe | 2.13(1.43-3.18) | <0.001 |
|  | Extremely severe | 5.97(3.53-10.09) | <0.001 |
|  | Presented to ED | 6.44(2.65-15.67) | <0.001 |
| Oral antivirals used for COVID-19 | No (ref) | 1.21(0.67-2.18) | 0.53 |
| Steroid inhalers used for COVID-19 | No (ref) | 2.42(1.29-4.54) | 0.006 |
| Monoclonal antibodies used for COVID-19 | No (ref) | 3.98(1.78-8.89) | 0.001 |
| Other medications used for COVID-19 | No (ref) | 0.70(0.32-1.56) | 0.39 |

# **Table S8. Distribution of data on the use of steroid inhalers, monoclonal antibodies, and oral antivirals among participants with Long COVID and those without Long COVID, by severity of acute COVID-19**

|  |  | Long COVID | |  |  | Long COVID | |  |  | Long COVID | |  |
| --- | --- | --- | --- | --- | --- | --- | --- | --- | --- | --- | --- | --- |
| Severity of Acute COVID-19 | Use of steroid inhalers | Yes  n=234 | No  n=953 | Total  n=1,187 | Use of monoclonal antibodies | Yes  n=234 | No  n=953 | Total  n=1,187 | Use of Antivirals | Yes  n=234 | No  n=953 | Total  n=1,187 |
| Mild illness |  | n=39 | n=377 | n=416 |  | n=39 | n=377 | n=416 |  | n=39 | n=377 | n=416 |
|  | Yes | 5(12.8%) | 8(2.1%) | 13(3.1%) | Yes | 6(15.4%) | 8(2.1%) | 14 (3.4%) | Yes | 5(12.8%) | 34(9.0%) | 39(9.4%) |
|  | No | 34(87.2%) | 369(97.9%) | 403(96.9%) | No | 33(83.6%) | 369(97.9%) | 402(96.6%) | No | 34(87.2%) | 343(91.0%) | 377(90.6%) |
| Moderately severe illness |  | n=127 | n=493 | n=620 |  | n=127 | n=493 | n=620 |  | n=127 | n=493 | n=620 |
|  | Yes | 16(12.6%) | 23(4.7%) | 39(6.3%) | Yes | 13(10.2%) | 20 (4.1%) | 33(5.3%) | Yes | 13(10.2%) | 37(7.5%) | 50(8.1%) |
|  | No | 111(87.4%) | 470(95.3%) | 581(93.7%) | No | 114(89.8%) | 473 (95.9%) | 587(94.7%) | No | 114(89.8%) | 456(92.5%) | 570(91.9%) |
| *Extremely severe and presented to ED |  | n=68 | n=83 | n=151 |  | n=68 | n=83 | n=151 |  | n=68 | n=83 | n=151 |
|  | Yes | 11(16.2%) | 6(7.2%) | 11(11.3%) | Yes | 18(26.5%) | 5 (6.0%) | 23(15.2%) | Yes | 19(27.9% | 13(15.7%) | 32(21.2%) |
|  | No | 57(83.8%) | 77(92.8%) | 134(99.7%) | No | 50(73.5%) | 78 (94.0%) | 128(84.8%) | No | 49(72.1%) | 70(84.3%) | 119(78.8%) |

*Because of small number of participants who presented to ED, extremely severe and presented to ED were combined.

# **Table S9. Distribution of data on the use of steroid inhalers, monoclonal antibodies, and oral antivirals among participants with Long COVID and those without Long COVID, by status of pre-existing comorbidity**

|  |  | With Long COVID | |  |  | With Long COVID | |  |  | With Long COVID | |  |
| --- | --- | --- | --- | --- | --- | --- | --- | --- | --- | --- | --- | --- |
| Pre-existing | Use of steroid inhalers | Yes | No | Total | Use of monoclonal antibodies | Yes | No | Total | Use of Antivirals | Yes | No | Total |
| comorbidity |  | n=234 | n=953 | n=1,187 |  | n=234 | n=953 | n=1,187 |  | n=234 | n=953 | n=1,187 |
| No comorbidity |  | n=70 | n=428 | n=498 |  | n=70 | n=428 | n=498 |  | n=70 | n=428 | n=498 |
|  | Yes | 12(17.1%) | 19(4.4%) | 31(6.2%) | Yes | 8(11.4%) | 14(3.3%) | 22(4.4%) | Yes | 5(7.1%) | 18(4.2%) | 23(4.6%) |
|  | No | 58(82.9%) | 409(95.6%) | 467(93.8%) | No | 62(88.6%) | 414(96.7%) | 476(95.6%) | No | 65(92.9%) | 410(95.8%) | 475(95.4%) |
| 1 comorbidity |  | n=72 | n=280 | n=352 |  | n=72 | n=280 | n=352 |  | n=72 | n=280 | n=352 |
|  | Yes | 12(16.7%) | 12(4.3%) | 24(6.8%) | Yes | 16(22.2%) | 9(3.2%) | 25(7.1%) | Yes | 14(19.4%) | 21(7.5%) | 35(9.9%) |
|  | No | 60(83.3%) | 268(95.7%) | 328(93.2%) | No | 56(77.8%) | 271(96.8%) | 327(92.9%) | No | 58(80.6%) | 259(92.5%) | 317(90.1%) |
| ≥ 2 comorbidities |  | n=92 | n=245 | n=337 |  | n=92 | n=245 | n=337 |  | n=92 | n=245 | n=337 |
|  | Yes | 8(8.7%) | 6(2.5%) | 14(4.2%) | Yes | 13(14.1%) | 10(4.1%) | 23(6.8%) | Yes | 18(19.6%) | 45(18.4%) | 63(18.7%) |
|  | No | 84(91.3%) | 239(97.5%) | 323(95.8%) | No | 79(85.9%) | 235(95.9%) | 314(93.2%) | No | 74(80.4%) | 200(81.6%) | 274(81.3%) |

# **Table S10. Subgroup analysis by severity of acute COVID-19, using the full model**

| Variable | Mild illness  n=416 | p-value | Moderately severe illness  n=620 | p-value | Extremely severe and presented to ED  n=151 | p-value |
| --- | --- | --- | --- | --- | --- | --- |
|  | adOR (95% CI) |  | adOR (95% CI) |  | adOR (95% CI) |  |
| Age | 0.98(0.94-1.01) | 0.15 | 0.99(0.97-1.01) | 0.29 | 1.00(0.97-1.03) | 0.93 |
| Female gender Male (ref) | 1.50(0.61-3.67) | 0.38 | 2.13(1.24-3.68) | 0.006 | 1.42(0.58-3.44) | 0.44 |
| Employed Unemployed (ref) | 0.81(0.32-2.07) | 0.66 | 1.16(0.66-2.02) | 0.61 | 2.36(0.98-5.69) | 0.06 |
| Owned house No (ref) | 0.81(0.34-1.92) | 0.63 | 0.99(0.63-1.55) | 0.96 | 0.87(0.35-2.13) | 0.76 |
| Born in Australia No (ref) | 1.51(0.57-4.02) | 0.41 | 1.08(0.63-1.84) | 0.78 | 1.12(0.42-2.94) | 0.82 |
| Education level Primary (ref) | - | - | - | - | - | - |
| High school | 0.49(0.20-1.19) | 0.12 | 0.30(0.04-2.13) | 0.23 | 0.73(0.30-1.74) | 0.47 |
| TAFE/Tertiary | - | - | 0.60(0.09-4.06) | 0.60 | - | - |
| Type of accommodation House (ref) | - | - | - | - | - | - |
| Unit/townhouse/other | 3.60(1.29-10.06) | 0.01 | 0.75(0.48-1.17) | 0.21 | 1.10(0.43-2.81) | 0.84 |
| Being in good health status No (ref) | 1.25(0.39-3.99) | 0.71 | 0.66(0.37-1.17) | 0.15 | 0.98(0.37-2.58) | 0.97 |
| Agreed to use face mask No (ref) | 3.35(0.99-11.38) | 0.05 | 0.64(0.34-1.21) | 0.17 | 0.97(0.27-3.43) | 0.96 |
| Vaccination status 0-2 doses (ref) | - | - | - | - | - | - |
| ≥3 doses | 0.62(0.25-1.54) | 0.30 | 0.91(0.55-1.50) | 0.70 | 1.24(0.46-3.36) | 0.67 |
| At least one comorbidity pre-existed No (ref) | 2.18(0.95-5.02) | 0.07 | 2.03(1.28-3.22) | 0.003 | 2.33(0.98-5.54) | 0.06 |
| Omicron as cause of the illness No (ref) | 7.01(1.51-32.63) | 0.01 | 0.98(0.56-1.71) | 0.94 | 1.79(0.69-4.69) | 0.23 |
| Number of times with COVID-19 | 2.13(0.93-4.90) | 0.07 | 1.28(0.83-1.95) | 0.26 | 0.85(0.44-1.64) | 0.62 |
| Oral antivirals used for COVID-19 No (ref) | 0.58(0.11-3.20) | 0.53 | 1.37(0.59-3.20) | 0.47 | 0.81(0.25-2.61) | 0.72 |
| Steroid inhalers used for COVID-19 No (ref) | 7.97(1.19-53.33) | 0.03 | 2.32(1.06-5.09) | 0.04 | 1.87(0.46-7.65) | 0.38 |
| Monoclonal antibodies used for COVID-19 No (ref) | 45.17(4.97-410.94) | 0.001 | 2.57(0.87-7.55) | 0.09 | 6.43(1.25-33.23) | 0.03 |

# **Table S11. Subgroup analysis by status of pre-existing comorbidity, using the full model**

| Variable | No comorbidity | p-value | 1 comorbidity | p-value | ≥ 2 comorbidities | p-value |
| --- | --- | --- | --- | --- | --- | --- |
|  | adOR (95% CI)  n=498 | | adOR (95% CI)  n=352 | | adOR (95% CI)  n=337 | |
| Age | 0.98(0.96-1.01) | 0.12 | 0.98(0.96-1.01) | 0.14 | 1.00(0.98-1.02) | 0.94 |
| Female gender Male (ref) | 1.28(0.64-2.57) | 0.48 | 1.29(0.60-2.74) | 0.52 | 2.45(1.24-4.84) | 0.01 |
| Employed Unemployed (ref) | 1.20(0.58-2.50) | 0.63 | 1.47(0.67-3.26) | 0.34 | 1.71(0.84-3.49) | 0.14 |
| Owned house No (ref) | 1.58(0.84-2.97) | 0.15 | 0.67(0.34-1.34) | 0.26 | 0.72(0.38-1.35) | 0.30 |
| Born in Australia No (ref) | 0.73(0.37-1.44) | 0.36 | 1.36(0.59-3.13) | 0.47 | 1.21(0.56-2.59) | 0.63 |
| Education level Primary (ref) | - | - | - | - | - | - |
| High school | 0.99(0.52-1.91) | 0.99 | 0.12(0.01-1.69) | 0.12 | 0.46(0.04-5.60) | 0.54 |
| TAFE/Tertiary | - | - | 0.24(0.02-3.18) | 0.28 | 1.35(0.12-15.51) | 0.81 |
| Type of accommodation House (ref) | - | - | - | - | - | - |
| Unit/townhouse/other | 1.10(0.59-2.07) | 0.76 | 1.32(0.62-2.81) | 0.47 | 0.79(0.43-1.45) | 0.45 |
| Being in good health status No (ref) | 0.71(0.26-1.95) | 0.51 | 0.65(0.28-1.50) | 0.31 | 1.02(0.52-2.01) | 0.94 |
| Agreed to use face mask No (ref) | 1.24(0.47-3.23) | 0.67 | 0.77(0.33-1.79) | 0.54 | 0.68(0.27-1.70) | 0.41 |
| Vaccination status 0-2 doses (ref) | - | - | - | - | - | - |
| ≥3 doses | 1.00(0.51-1.93) | 1.00 | 1.18(0.55-2.53) | 0.67 | 0.57(0.26-1.23) | 0.15 |
| Omicron as cause of the illness No (ref) | 1.73(0.79-3.81) | 0.17 | 1.15(0.51-2.57) | 0.74 | 1.56(0.70-3.49) | 0.28 |
| Severity of acute COVID-19 Mild (ref) |  |  |  |  |  |  |
| Moderately severe | 1.75(0.90-3.39) | 0.10 | 3.14(1.42-6.93) | 0.005 | 1.96(0.95-4.03) | 0.07 |
| Extremely severe | 5.09(2.04_12.71) | 0.001 | 7.18(2.60-19.86) | <0.001 | 8.54(3.29-22.16) | <0.001 |
| Presented to ED | 3.97(0.49-32.21) | 0.20 | 25.36(3.63-176.92) | 0.001 | 5.12(1.37-19.13) | 0.02 |
| Number of times with COVID-19 | 1.35(0.81-2.23) | 0.25 | 1.44(0.80-2.57) | 0.22 | 0.73(0.37-1.44) | 0.37 |
| Oral antivirals used for COVID-19 No (ref) | 0.70(0.17-2.90) | 0.62 | 3.44(1.14-10.38) | 0.03 | 0.98(0.39-2.52) | 0.98 |
| Steroid inhalers used for COVID-19 No (ref) | 3.31(1.18-9.30) | 0.02 | 1.48(0.46-4.73) | 0.51 | 3.51(0.89-13.88) | 0.07 |
| Monoclonal antibodies used for COVID-19 No (ref) | 1.59(0.39-6.49) | 0.52 | 2.97(0.68-12.98) | 0.15 | 9.71(2.10-44.77) | 0.004 |
| Other medications used for COVID-19 No (ref) | 1.33(0.28-6.30) | 0.72 | 1.61(0.40-6.52) | 0.51 | 0.17(0.04-0.78) | 0.02 |

# **Table S12. Long-COVID symptoms by Omicron variant vs. pre-Omicron variants (mainly Delta)**

| Symptoms | Omicron  (Participants= 968), n(%) | pre-Omicron  (Participants=237), n(%) |
| --- | --- | --- |
| Tiredness | 179 (18.5%) | 44 (18.6%) |
| Shortness of breath | 111 (11.5%) | 33 (13.9%) |
| Weakness | 108 (11.2%) | 33 (13.9%) |
| Dizziness | 67 (6.9%) | 22 (9.3%) |
| Joint pains | 57 (5.9%) | 21 (8.9%) |
| Feeling faint | 60 (6.2%) | 18 (7.6%) |
| Brain fog | 94 (9.7%) | 17 (7.2%) |
| Muscle aches | 77 (8.0%) | 15 (6.3%) |
| **Palpitations | 16 (1.7%) | 15 (6.3%) |
| Difficulty in concentrating | 78 (8.1%) | 14 (5.9%) |
| Mood swings | 29 (3.0%) | 13 (5.5%) |
| Memory loss | 49 (5.1%) | 12 (5.1%) |
| Chest pain | 33 (3.4%) | 12 (5.1%) |
| Stomach pains | 35 (3.6%) | 12 (5.1%) |
| Depression | 36 (3.7%) | 10 (4.2%) |
| Loss of smell or taste | 7 (0.7%) | 0 |
| Trembling/shaking | 21 (2.2%) | 9 (3.8%) |
| Cough | 20 (2.1%) | 6 (2.5%) |
| Headache | 9 (0.9%) | 4 (1.7%) |
| *Hallucinations | 1 (0.1%) | 3 (1.3%) |
| *p-value<0.05, **p-value<0.003 |  |  |

# **Clustering Results**

The main text presents the primary results of the Partitioning Around Medoids (PAM) clustering method. This Supplementary text presents additional results, for both the PAM clustering and Hierarchical Clustering (HC) methods. Both methods yielded similar descriptive and statistical results.

## *Partitioning Around Medoids (PAM) Clustering Results*


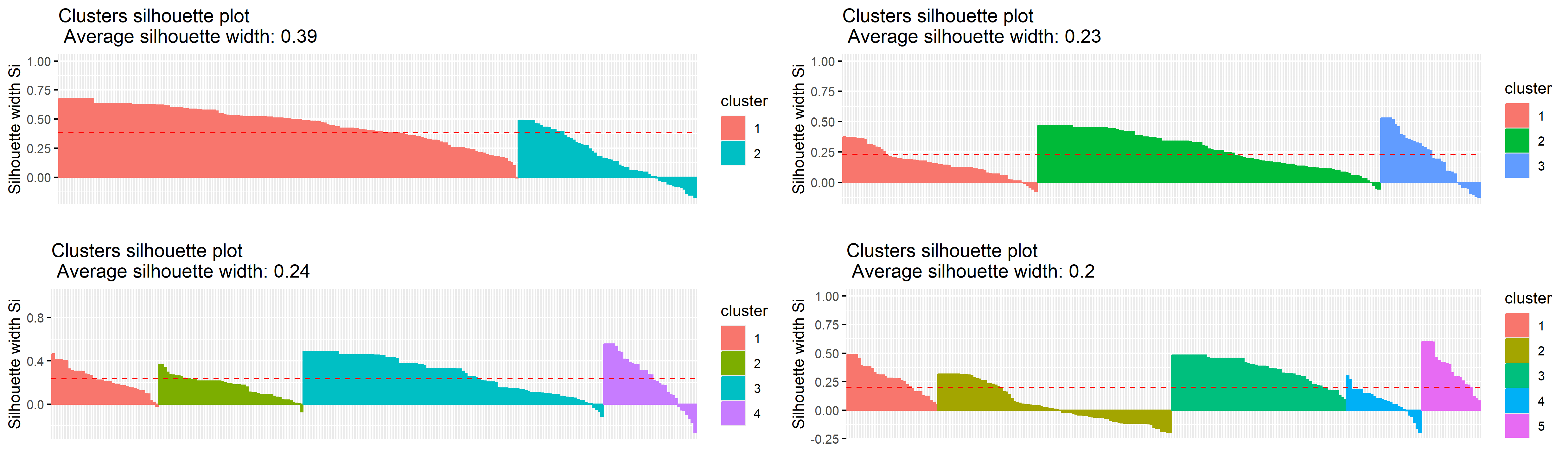


### Figure S5. Silhouette plot for two to five clusters using the PAM clustering method.


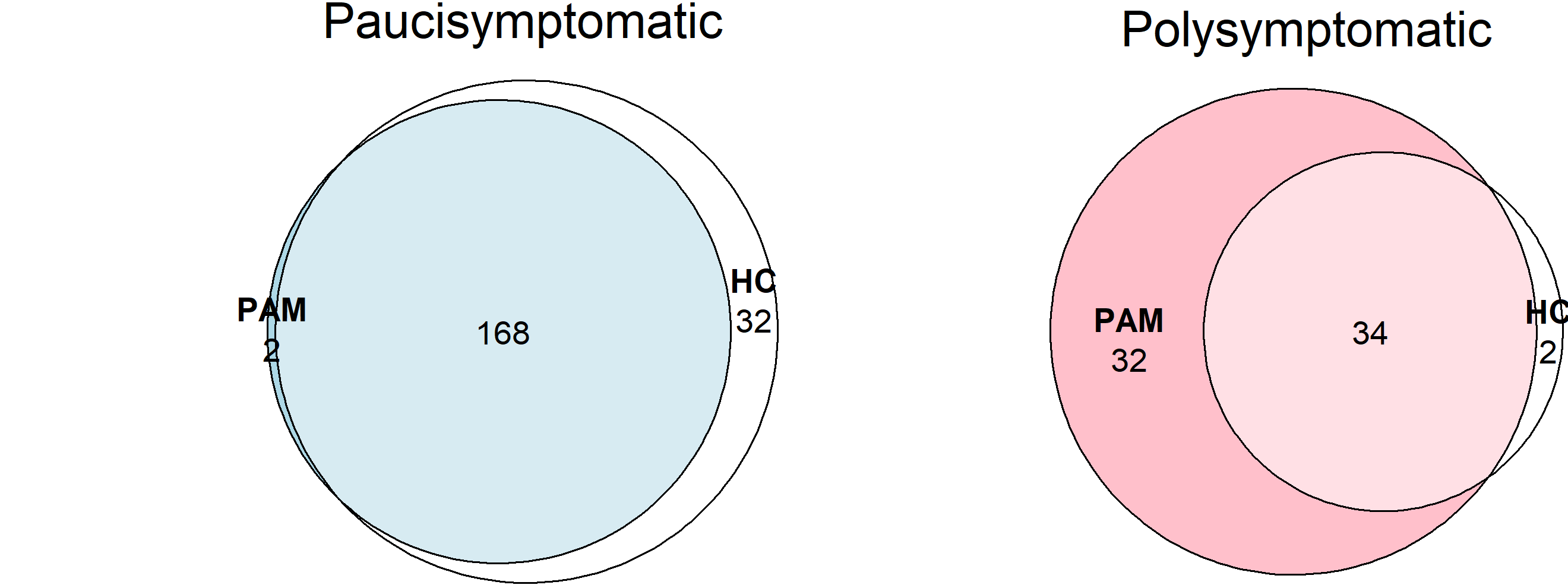


### Figure S6. Euler plots comparing the pauci-symptomatic and polysymptomatic clusters obtained by PAM and Hierarchical Clustering methods.

### Table S13. Baseline participant characteristics by cluster (Total Participants = 236) using PAM clustering.

| Variable | | Paucisymptomatic Cluster (Participants = 170), n (%) | Polysymptomatic Cluster (Participants = 66), n (%) | Test Statistic* | P-value |
| --- | --- | --- | --- | --- | --- |
| Age, median (IQR) | | 36.00 (27.00-49.50) | 39.50 (27.25-53.00) | 0.99 | 0.32 |
| Gender | |  |  | 4.91 | 0.03 |
|  | Male | 53 (31.18) | 10 (15.15) |  |  |
|  | Female | 115 (67.65) | 53 (80.3) |  |  |
|  | Non-binary | 2 (1.18) | 3 (4.55) |  |  |
| Language spoken as a child | |  |  | 0.00 | 0.99 |
|  | English | 156 (91.76) | 60 (92.31) |  |  |
|  | Not English | 14 (8.24) | 5 (7.69) |  |  |
| Employment | |  |  | 2.80 | 0.09 |
|  | Unemployed | 37 (21.76) | 22 (33.33) |  |  |
|  | Employed | 133 (78.24) | 44 (66.67) |  |  |
| State | |  |  | 4.02 | 0.78 |
|  | Australian Capital Territory | 27 (15.88) | 10 (15.15) |  |  |
|  | New South Wales | 23 (13.53) | 6 (9.09) |  |  |
|  | Northern Territory | 11 (6.47) | 4 (6.06) |  |  |
|  | Queensland | 20 (11.76) | 9 (13.64) |  |  |
|  | South Australia | 21 (12.35) | 7 (10.61) |  |  |
|  | Tasmania | 20 (11.76) | 8 (12.12) |  |  |
|  | Victoria | 27 (15.88) | 8 (12.12) |  |  |
|  | Western Australia | 21 (12.35) | 14 (21.21) |  |  |
| Homeowner | |  |  | 2.79 | 0.1 |
|  | Not homeowner | 63 (37.06) | 33 (50) |  |  |
|  | Homeowner | 107 (62.94) | 33 (50) |  |  |
| Country of Birth | |  |  | 5.33 | 0.02 |
|  | Other Country | 23 (13.53) | 18 (27.27) |  |  |
|  | Australia | 147 (86.47) | 48 (72.73) |  |  |
| Education | |  |  | 0.00 | 0.96 |
|  | Primary School | 1 (0.59) | 2 (3.03) |  |  |
|  | High School | 32 (18.82) | 13 (19.7) |  |  |
|  | TAFE / Tertiary | 137 (80.59) | 51 (77.27) |  |  |
| Accommodation | |  |  | 0.76 | 0.38 |
|  | House | 120 (70.59) | 51 (77.27) |  |  |
|  | Unit/Townhouse/Other | 50 (29.41) | 15 (22.73) |  |  |
| Health before COVID-19 | |  |  | 5.33 | 0.02 |
|  | Poor | 23 (13.53) | 18 (27.27) |  |  |
|  | Good | 147 (86.47) | 48 (72.73) |  |  |
| Uses mask in public | |  |  | 0.01 | 0.92 |
|  | Disagree | 118 (69.41) | 44 (67.69) |  |  |
|  | Agree | 52 (30.59) | 21 (32.31) |  |  |
| Vaccination doses | |  |  | 0.01 | 0.9 |
|  | 0-2 doses | 40 (24.69) | 17 (26.56) |  |  |
|  | At least 3 doses | 122 (75.31) | 47 (73.44) |  |  |
| Any pre-existing comorbidity | |  |  | 2.34 | 0.13 |
|  | No pre-existing comorbidity | 55 (32.35) | 14 (21.21) |  |  |
|  | Any pre-existing comorbidity | 115 (67.65) | 52 (78.79) |  |  |
| SARS-CoV-2 variant | |  |  | 1.53 | 0.22 |
|  | Other variant | 40 (23.53) | 10 (15.15) |  |  |
|  | Omicron | 130 (76.47) | 56 (84.85) |  |  |
| Number of times with COVID-19 | |  |  | 2.98 | 0.08 |
|  | Once | 122 (71.76) | 54 (81.82) |  |  |
|  | Twice | 34 (20) | 11 (16.67) |  |  |
|  | ≥ 3 times | 14 (8.24) | 1 (1.52) |  |  |
| Severity of acute COVID-19 infection | |  |  | 22.73 | 0.001 |
|  | Mild | 31 (18.24) | 7 (10.61) |  |  |
|  | Moderately Severe | 102 (60) | 26 (39.39) |  |  |
|  | Extremely severe | 24 (14.12) | 28 (42.42) |  |  |
|  | Presented to ED | 13 (7.65) | 5 (7.58) |  |  |
| Antiviral treatment for COVID-19 | |  |  | 4.29 | 0.04 |
|  | No antiviral treatment | 135 (81.82) | 60 (93.75) |  |  |
|  | Antiviral treatment | 30 (18.18) | 4 (6.25) |  |  |
| Steroid inhaler treatment for COVID-19 | |  |  | 0.00 | 0.99 |
|  | No steroid inhaler treatment | 102 (60) | 40 (60.61) |  |  |
|  | Steroid inhaler treatment | 68 (40) | 26 (39.39) |  |  |
| Monoclonal antibody treatment for COVID-19 | |  |  | 15.43 | 0.001 |
|  | No Monoclonal antibody treatment | 133 (78.24) | 66 (100) |  |  |
|  | Monoclonal antibody treatment | 37 (21.76) | 0 (0) |  |  |
| Other medications for COVID-19 | |  |  | 0.11 | 0.74 |
|  | No other medication | 119 (70.83) | 46 (74.19) |  |  |
|  | Other medication | 49 (29.17) | 16 (25.81) |  |  |
| *Kruskal-Wallis test was applied to continuous variables and ordinal variables. Pearson’s chi-squared was applied to categorical variables (categories with less than 5 counts were excluded). This table provides a summary of baseline characteristics of the 236 participants assigned to the paucisymptomatic (170 patients) and polysymptomatic (66 patients) clusters via the partitioning around medoids clustering method. Values are presented as n (%), denoting counts and percentages of patients within each cluster, except for age, which is presented as median (IQR). | | | | | |

###
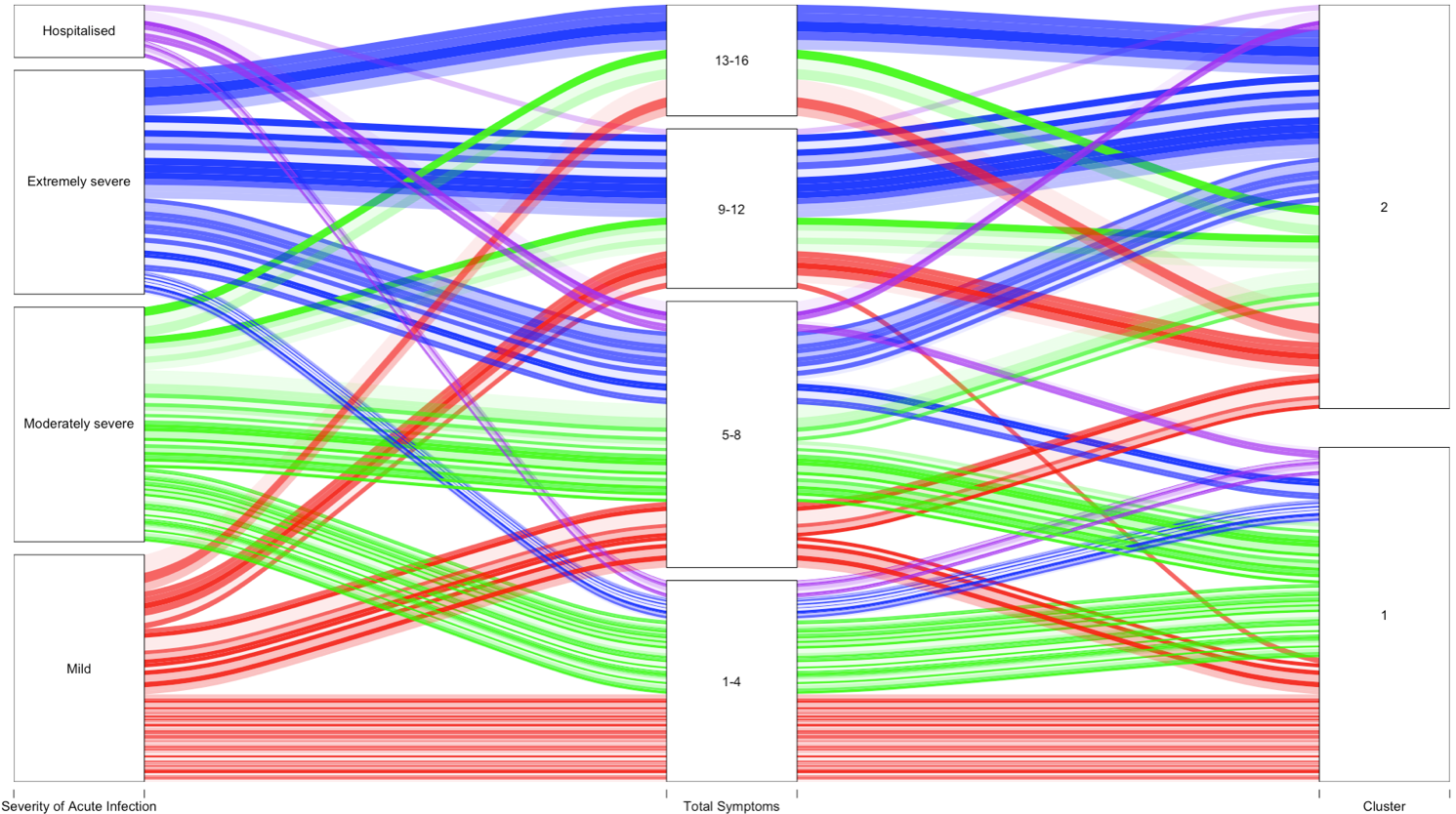
Figure S7. Alluvial plot of severity of acute COVID-19 infection with total number of long-COVID symptoms experienced by participants and long-COVID cluster membership using the PAM clustering method.

##

### Table S14. Participant functional correlates following COVID-19 infection by cluster membership (Total Participants = 236) by PAM clustering.

| Variable | | Pauci-symptomatic Cluster  (Participants = 170), n (%) | Polysymptomatic Cluster (Participants = 66), n (%) | Test Statistic* | P-value |
| --- | --- | --- | --- | --- | --- |
| Number of symptoms, median (IQR) | | 3 (1-4) | 9 (7-11) | 137.13 | 0.001 |
| Duration of symptoms, median (IQR) | | 3 (2-5) | 3 (2-5) | 0.8 | 0.37 |
| Performing activities (moving table) | |  |  | 14.12 | 0.001 |
|  | Limited | 67 (39.41) | 44 (66.67) |  |  |
|  | Not Limited | 103 (60.59) | 22 (33.33) |  |  |
| Performing activities (climbing stairs) | | | | 9.59 | 0.002 |
|  | Limited | 83 (48.82) | 47 (71.21) |  |  |
|  | Not Limited | 87 (51.18) | 19 (28.79) |  |  |
| How often felt calm and peaceful | | | | 12.66 | 0.001 |
|  | Little/none of the time | 39 (22.94) | 30 (45.45) |  |  |
|  | Some of the time | 74 (43.53) | 25 (37.88) |  |  |
|  | All/most of the time | 57 (33.53) | 11 (16.67) |  |  |
| How often felt they had lots of energy | | | | 21 | 0.001 |
|  | Little/none of the time | 56 (32.94) | 44 (66.67) |  |  |
|  | Some of the time | 86 (50.59) | 18 (27.27) |  |  |
|  | All/most of the time | 28 (16.47) | 4 (6.06) |  |  |
| How often felt downhearted | | |  | 1.37 | 0.24 |
|  | Little/none of the time | 63 (37.06) | 20 (30.3) |  |  |
|  | Some of the time | 71 (41.76) | 28 (42.42) |  |  |
|  | All/most of the time | 36 (21.18) | 18 (27.27) |  |  |
| How often physical or emotional problems interfered with social activities | | | | 4.99 | 0.03 |
|  | Little/none of the time | 60 (35.29) | 16 (24.24) |  |  |
|  | Some of the time | 76 (44.71) | 28 (42.42) |  |  |
|  | All/most of the time | 34 (20) | 22 (33.33) |  |  |
| Accomplished less with work or other activities | | | | 13.0 | 0.001 |
|  | Yes | 54 (31.76) | 35 (53.03) |  |  |
|  | Maybe | 45 (26.47) | 19 (28.79) |  |  |
|  | No | 71 (41.76) | 12 (18.18) |  |  |
| Worked less carefully | |  |  | 9.03 | 0.003 |
|  | Yes | 42 (24.71) | 30 (45.45) |  |  |
|  | Maybe | 48 (28.24) | 16 (24.24) |  |  |
|  | No | 80 (47.06) | 20 (30.3) |  |  |
| How often pain interfered with work | | | | 7.53 | 0.006 |
|  | Little/no interference | 122 (71.76) | 36 (54.55) |  |  |
|  | Moderate interference | 28 (16.47) | 13 (19.7) |  |  |
|  | A lot of interference | 20 (11.76) | 17 (25.76) |  |  |
| Unable to work due to symptoms in last 4 weeks | | | | 0.86 | 0.35 |
|  | Yes | 66 (38.82) | 30 (45.45) |  |  |
|  | No | 104 (61.18) | 36 (54.55) |  |  |
| Self-reported health rating post-COVID, n (%) | | | | 25.28 | 0.001 |
|  | Poor | 6 (3.53) | 12 (18.18) |  |  |
|  | Fair | 60 (35.29) | 34 (51.52) |  |  |
|  | Good | 61 (35.88) | 16 (24.24) |  |  |
|  | Very Good | 27 (15.88) | 4 (6.06) |  |  |
|  | Excellent | 16 (9.41) | 0 (0) |  |  |
| Self-reported health rating difference pre/post COVID | | | | 5.50 | 0.02 |
|  | Improved | 2 (1.18) | 1 (1.52) |  |  |
|  | Same | 87 (51.18) | 22 (33.33) |  |  |
|  | Worse | 81 (47.65) | 43 (65.15) |  |  |

*Kruskal-Wallis test was applied to continuous variables and ordinal variables. Pearson’s chi-squared was applied to categorical variables (categories with less than 5 counts were excluded). For variables with two categories, phi was computed as effect size, while for variables with more than two categories, Cramer’s V was used.

This table provides a summary of baseline characteristics of the 236 participants assigned to the paucisymptomatic (170 patients) and polysymptomatic (66 patients) clusters via the partitioning around medoids clustering method. Values are presented as n (%), denoting counts and percentages of patients within each cluster, except for Number of Symptoms and Duration of Symptoms, which are presented as median (IQR).

## **Hierarchical Clustering Results**

### *Description of Results*

Agglomerative hierarchical clustering was performed, comparing different distance matrices (Jaccard, Manhattan, Gower) and linkage functions (Complete, Average). Silhouette scores and a scree plot were used to identify the optimal number of clusters (Figures S8, S9). After comparing resultant dendrograms and Silhouette scores, the Manhattan distance measure and Complete linkage function were chosen. The average Silhouette score was 0.40.

Hierarchical agglomerative clustering assigned 200 people to one cluster and 36 people to a second cluster (Figure S10). Baseline demographic and socioeconomic variables, and data relating to vaccination status, COVID-19 infection and treatment are presented for each cluster in Table S15. Pearson’s chi-squared tests and Kruskal-Wallis tests identified significant differences in gender (χ^2^=10.5, p=0.001), homeownership (χ^2^=8.38, p = 0.01), any pre-existing comorbidity (χ^2^=7.82, p=0.01), and treatment with monoclonal antibody (χ^2^=4.26, p=0.04) between the two clusters. The median symptom count per participant was 3 (IQR: 2-5) in the first cluster and 11 (9-13) in the second cluster (H=80.65, p=0.001) (Table S17), thus they are designated as pauci-symptomatic and polysymptomatic, respectively.

The full multivariate logistic regression model containing potential predictors of polysymptomatic cluster membership is presented in table S16. Using a p-value of 0.10, backward stepwise regression was used to obtain the final model (Figure S11). Female gender was nominally significantly associated with polysymptomatic cluster membership (9.01 [2.14-83.98], p=0.001), and having any pre-existing comorbidity (5.08 [1.74-19.99], p=0.01). Being born in Australia was associated with a protective effect (0.34 [0.13-0.86], p=0.02). Being a homeowner appeared to have a possible protective effect (0.50 [0.21-1.12], p=0.09), as did monoclonal antibody treatment (0.25 [0.02-1.26], p=0.098), however, the 95% confidence intervals included the null value (adjusted odds ratio of 1) for both variables. After applying Bonferroni correction, female gender remained significantly associated with polysymptomatic cluster membership.

There was no significant difference in the median duration of symptoms between clusters (3 [IQR: 2-5] months in the pauci-symptomatic cluster and 3 [2-5.75] months in the polysymptomatic cluster) (H=0.27, p=0.60) (Table S17).

In terms of functional correlates, membership of the polysymptomatic cluster was strongly associated with limitations in terms of physical activities (moving tables [H=16.05, p=0.001], climbing stairs [H=19.54, p=0.001]), emotional status (did not often feel calm, peaceful [H=10.8, p=0.001] or energetic [H=8.18, p=0.004], more often felt downhearted [H=4.84, p=0.03]), work impacts (accomplished less work [H=8.86, p=0.003], worked less carefully [H=5.67, p=0.02], pain interfered with work [H=4.15, p=0.04]), and health-related outcomes (more likely to self-report their health as poorer post-COVID compared to the pauci-symptomatic cluster [H=22.75, p=0.001], and to report decline in health post-COVID compared to baseline [H=5.88, p=0.02]) (Table S17). Overall, the results of hierarchical clustering also indicated a smaller, more severe, subgroup of long-COVID patients experiencing a greater number of symptoms and worse functional outcomes.

###
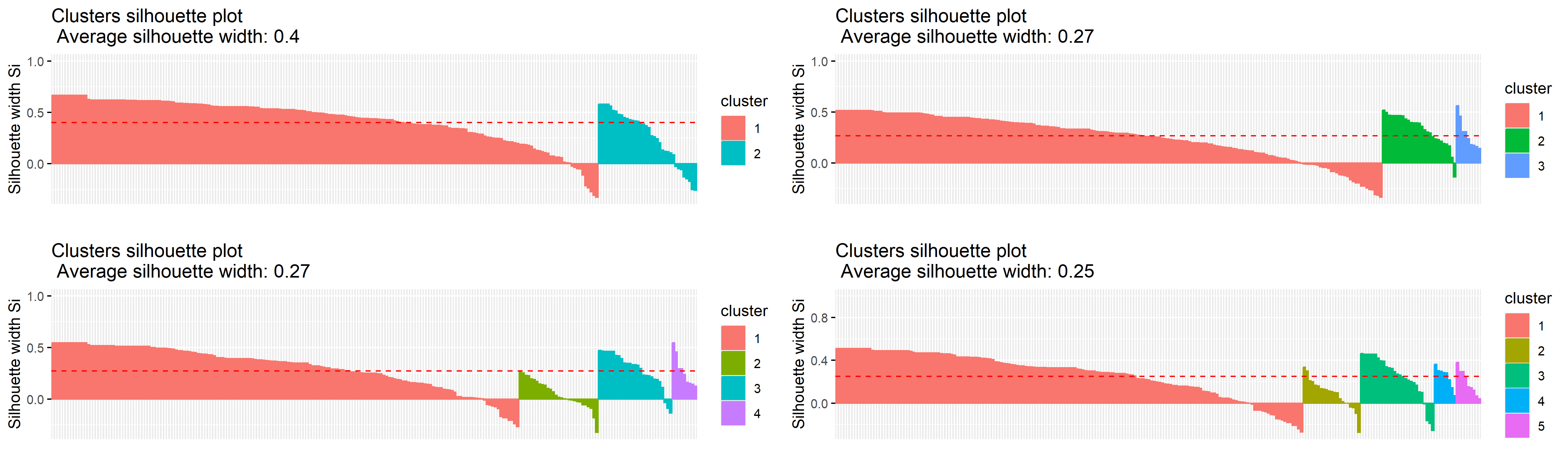
Figure S8. Silhouette plot for 2-5 clusters obtained by hierarchical clustering.


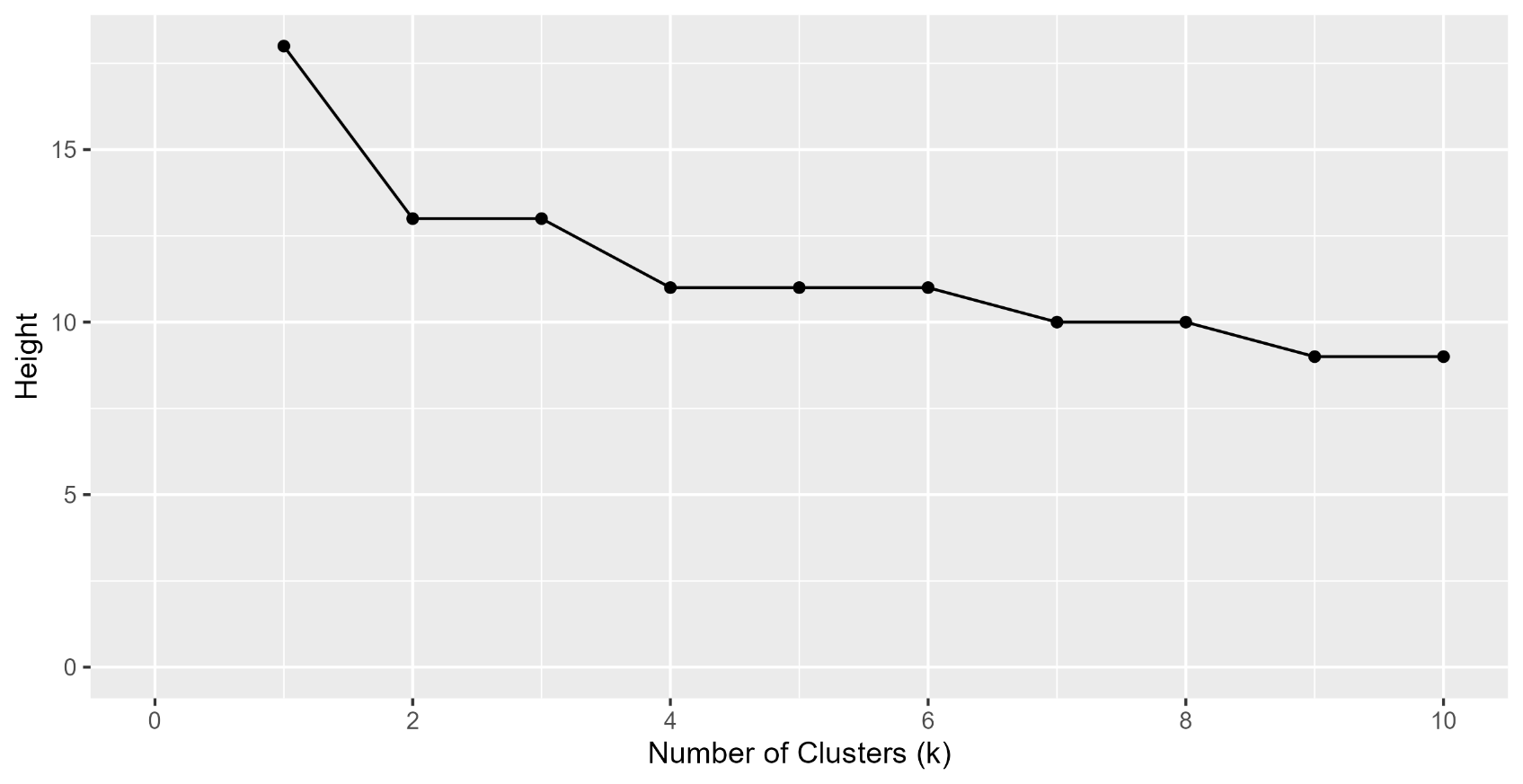


### Figure S9. Scree plot for hierarchical clustering.

The “elbow” of the plot occurs at k = 2, identifying this as the optimal number of clusters.


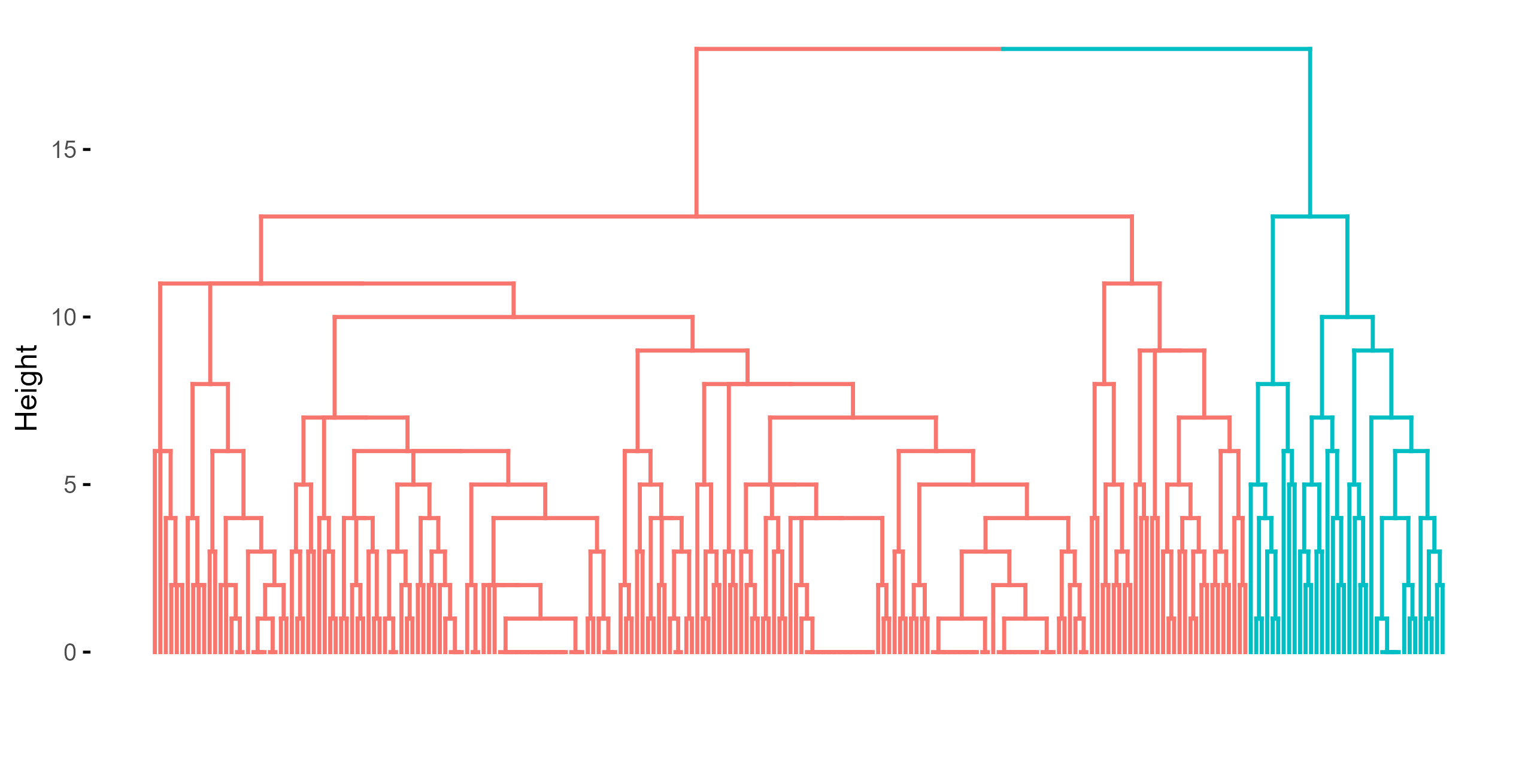


### Figure S10. Dendrogram plot depicting clusters obtained using Hierarchical clustering with Manhattan distance and complete linkage.

Data points belonging to the pauci-symptomatic cluster (n=200) are highlighted in red and data points belonging to the polysymptomatic cluster (n=36) are highlighted in blue.

### Table S15. Baseline participant characteristics by cluster (Total Participants = 236) using hierarchical clustering.

| Variable | | Paucisymptomatic Cluster (Participants = 200), n (%) | Polysymptomatic Cluster  (Participants = 36),  n (%) | Test Statistic* | P-value |
| --- | --- | --- | --- | --- | --- |
| Age, median (IQR) | | 37.00 (27.75-50.00) | 36.00 (25.75-52.50) | 0.00 | 0.99 |
| Gender | |  |  | 10.50 | 0.001 |
|  | Male | 62 (31) | 1 (2.78) |  |  |
|  | Female | 135 (67.5) | 33 (91.67) |  |  |
|  | Non-binary | 3 (1.5) | 2 (5.56) |  |  |
| Language spoken as a child | |  |  | 0.07 | 0.78 |
|  | English | 182 (91.46) | 34 (94.44) |  |  |
|  | Not English | 17 (8.54) | 2 (5.56) |  |  |
| Employment | |  |  | 0.39 | 0.53 |
|  | Unemployed | 48 (24) | 11 (30.56) |  |  |
|  | Employed | 152 (76) | 25 (69.44) |  |  |
| State | |  |  | 4.09 | 0.77 |
|  | Australian Capital Territory | 31 (15.5) | 6 (16.67) |  |  |
|  | New South Wales | 27 (13.5) | 2 (5.56) |  |  |
|  | Northern Territory | 12 (6) | 3 (8.33) |  |  |
|  | Queensland | 25 (12.5) | 4 (11.11) |  |  |
|  | South Australia | 25 (12.5) | 3 (8.33) |  |  |
|  | Tasmania | 23 (11.5) | 5 (13.89) |  |  |
|  | Victoria | 30 (15) | 5 (13.89) |  |  |
|  | Western Australia | 27 (13.5) | 8 (22.22) |  |  |
| Homeowner | |  |  | 8.38 | 0.01 |
|  | Not homeowner | 73 (36.5) | 23 (63.89) |  |  |
|  | Homeowner | 127 (63.5) | 13 (36.11) |  |  |
| Country of Birth | |  |  | 2.41 | 0.12 |
|  | Other Country | 31 (15.5) | 10 (27.78) |  |  |
|  | Australia | 169 (84.5) | 26 (72.22) |  |  |
| Education | |  |  | 0.01 | 0.9 |
|  | Primary School | 2 (1) | 1 (2.78) |  |  |
|  | High School | 39 (19.5) | 6 (16.67) |  |  |
|  | TAFE / Tertiary | 159 (79.5) | 29 (80.56) |  |  |
| Accommodation | |  |  | 1.92 | 0.17 |
|  | House | 141 (70.5) | 30 (83.33) |  |  |
|  | Unit/Townhouse/Other | 59 (29.5) | 6 (16.67) |  |  |
| Health before COVID-19 | |  |  | 1.15 | 0.28 |
|  | Poor | 32 (16) | 9 (25) |  |  |
|  | Good | 168 (84) | 27 (75) |  |  |
| Uses mask in public | |  |  | 0.27 | 0.61 |
|  | Disagree | 139 (69.85) | 23 (63.89) |  |  |
|  | Agree | 60 (30.15) | 13 (36.11) |  |  |
| Vaccination doses | |  |  | 0.16 | 0.69 |
|  | 0-2 doses | 47 (24.48) | 10 (29.41) |  |  |
|  | At least 3 doses | 145 (75.52) | 24 (70.59) |  |  |
| Any pre-existing comorbidity | |  |  | 7.82 | 0.01 |
|  | No pre-existing comorbidity | 66 (33) | 3 (8.33) |  |  |
|  | Any pre-existing comorbidity | 134 (67) | 33 (91.67) |  |  |
| SARS-CoV-2 variant | |  |  | 0.00 | 0.99 |
|  | Other variant | 42 (21) | 8 (22.22) |  |  |
|  | Omicron | 158 (79) | 28 (77.78) |  |  |
| Number of times with COVID-19 | |  |  | 0.49 | 0.48 |
|  | Once | 151 (75.5) | 25 (69.44) |  |  |
|  | Twice | 36 (18) | 9 (25) |  |  |
|  | ≥ 3 times | 13 (6.5) | 2 (5.56) |  |  |
|  | More than 4 times | 0 (0) | 0 (0) |  |  |
| Severity of acute COVID-19 infection | |  |  | 8.03 | 0.045 |
|  | Mild | 32 (16) | 6 (16.67) |  |  |
|  | Moderately Severe | 113 (56.5) | 15 (41.67) |  |  |
|  | Extremely severe | 38 (19) | 14 (38.89) |  |  |
|  | Presented to ED | 17 (8.5) | 1 (2.78) |  |  |
| Antiviral treatment for COVID-19 | |  |  | 1.94 | 0.16 |
|  | No antiviral treatment | 162 (83.51) | 33 (94.29) |  |  |
|  | Antiviral treatment | 32 (16.49) | 2 (5.71) |  |  |
| Steroid inhaler treatment for COVID-19 | |  |  | 0.64 | 0.42 |
|  | No steroid inhaler treatment | 123 (61.5) | 19 (52.78) |  |  |
|  | Steroid inhaler treatment | 77 (38.5) | 17 (47.22) |  |  |
| Monoclonal antibody treatment for COVID-19 | |  |  | 4.26 | 0.04 |
|  | No Monoclonal antibody treatment | 164 (82) | 35 (97.22) |  |  |
|  | Monoclonal antibody treatment | 36 (18) | 1 (2.78) |  |  |
| Other medications for COVID-19 | |  |  | 0.46 | 0.5 |
|  | No other medication | 137 (70.62) | 28 (77.78) |  |  |
|  | Other medication | 57 (29.38) | 8 (22.22) |  |  |
| *Kruskal-Wallis test was applied to continuous variables and ordinal variables. Pearson’s chi-squared was applied to categorical variables (categories with less than 5 counts were excluded). This table provides a summary of baseline characteristics of the 236 participants assigned to the paucisymptomatic (200 patients) and polysymptomatic (36 patients) clusters via the hierarchical clustering method. Values are presented as n (%), denoting counts and percentages of patients within each cluster, except for age, which is presented as median (IQR). | | | | | |

### Table S16. Full model containing all potential predictors of polysymptomatic cluster membership by hierarchical clustering.

| Variable | Adjusted Odds Ratio (95% CI) | P-value |
| --- | --- | --- |
| Age | 1.02 (0.98-1.06) | 0.436 |
| Gender: Female | 21.79 (2.36-3270.69) | 0.002 |
| Employment: Employed | 0.98 (0.32-3.29) | 0.968 |
| Homeowner | 0.53 (0.19-1.41) | 0.205 |
| Born in Australia | 0.35 (0.12-0.99) | 0.049 |
| Education: High School | 1.3 (0.06-30.07) | 0.863 |
| Education: TAFE/Tertiary | 1.07 (0.06-20.77) | 0.959 |
| Accommodation: Unit/Townhouse/Other | 0.92 (0.3-2.61) | 0.875 |
| Good health status | 0.8 (0.27-2.47) | 0.683 |
| Uses face mask in public | 2.67 (1.04-6.9) | 0.041 |
| Vaccine doses: ≥ 3 | 0.85 (0.3-2.55) | 0.767 |
| Any pre-existing comorbidity | 3.18 (0.96-13.56) | 0.058 |
| Variant: Omicron | 0.57 (0.18-1.9) | 0.346 |
| Times had COVID-19 | 1.34 (0.58-3.04) | 0.484 |
| Acute COVID-19 infection: Moderately Severe | 0.52 (0.15-1.86) | 0.302 |
| Acute COVID-19 infection: Extremely Severe | 1.24 (0.33-4.98) | 0.750 |
| Acute COVID-19 infection: Presented to ED | 0.47 (0.03-3.99) | 0.506 |
| Antiviral treatment | 0.84 (0.11-5.06) | 0.857 |
| Steroid inhaler treatment | 1.85 (0.73-4.81) | 0.193 |
| Monoclonal antibody treatment | 0.11 (0-1.56) | 0.111 |
| Other medication | 0.95 (0.3-2.78) | 0.930 |
|  | | |


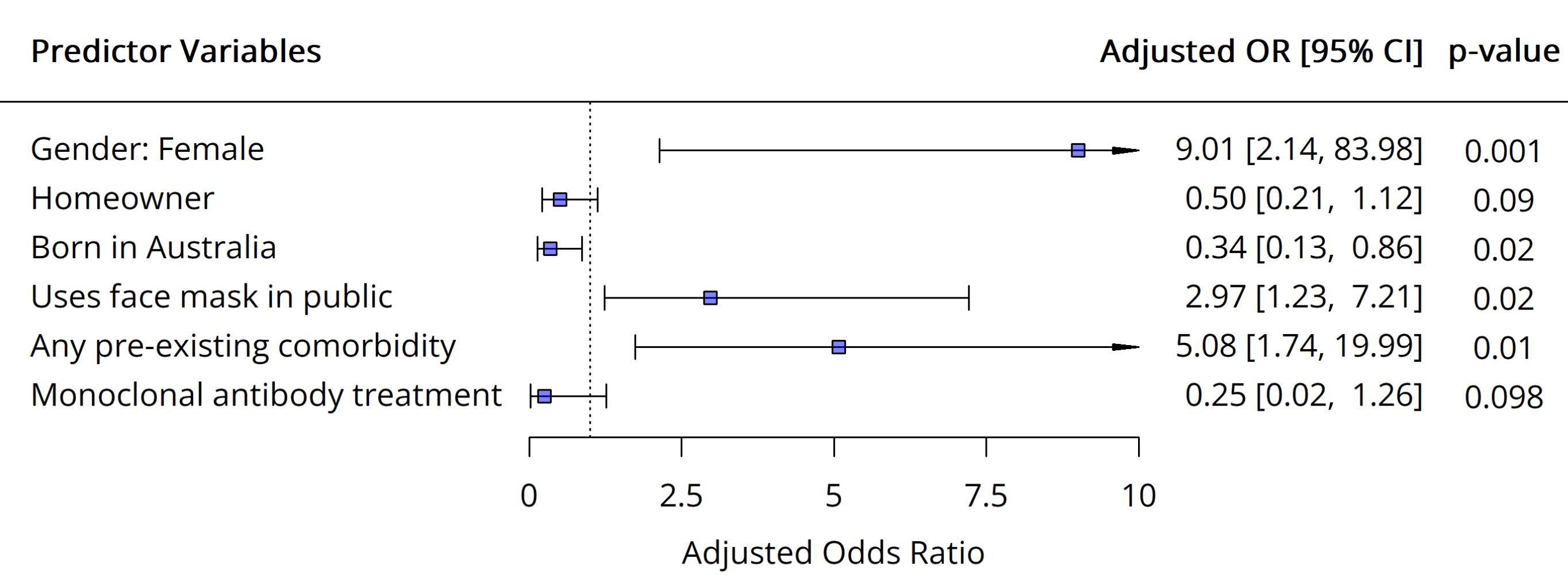


### Figure S11. Forest plot showing adjusted odds ratios and 95% confidence interval for predictors of polysymptomatic cluster membership by hierarchical clustering (Model 2).

Blue squares indicate the odds ratio and horizontal lines depict the 95% confidence interval (arrows shown where the confidence interval is truncated).

### Table S17. Participant functional correlates following COVID-19 infection by cluster membership (Total Participants = 236) by hierarchical clustering.

| Variable | | Pauci-symptomatic Cluster  (Participants = 200), n (%) | Polysymptomatic Cluster (Participants = 36),  n (%) | Test Statistic* | P-value |
| --- | --- | --- | --- | --- | --- |
| Number of symptoms, median (IQR) | | 3 (2-5) | 11 (9-13) | 80.65 | 0.001 |
| Duration of symptoms, median (IQR) | | 3 (2-5) | 3 (2-5.75) | 0.27 | 0.60 |
| Performing activities (moving table) | |  |  | 16.05 | 0.001 |
|  | Limited | 83 (41.5) | 28 (77.78) |  |  |
|  | Not Limited | 117 (58.5) | 8 (22.22) |  |  |
| Performing activities (climbing stairs) | | | | 19.54 | 0.001 |
|  | Limited | 98 (49) | 32 (88.89) |  |  |
|  | Not Limited | 102 (51) | 4 (11.11) |  |  |
| How often felt calm and peaceful | | | | 10.8 | 0.001 |
|  | Little/none of the time | 50 (25) | 19 (52.78) |  |  |
|  | Some of the time | 87 (43.5) | 12 (33.33) |  |  |
|  | All/most of the time | 63 (31.5) | 5 (13.89) |  |  |
| How often felt they had lots of energy | | | | 8.18 | 0.004 |
|  | Little/none of the time | 77 (38.5) | 23 (63.89) |  |  |
|  | Some of the time | 93 (46.5) | 11 (30.56) |  |  |
|  | All/most of the time | 30 (15) | 2 (5.56) |  |  |
| How often felt downhearted | | |  | 4.84 | 0.03 |
|  | Little/none of the time | 74 (37) | 9 (25) |  |  |
|  | Some of the time | 86 (43) | 13 (36.11) |  |  |
|  | All/most of the time | 40 (20) | 14 (38.89) |  |  |
| How often physical or emotional problems interfered with social activities | | | | 10.17 | 0.001 |
|  | Little/none of the time | 72 (36) | 4 (11.11) |  |  |
|  | Some of the time | 86 (43) | 18 (50) |  |  |
|  | All/most of the time | 42 (21) | 14 (38.89) |  |  |
| Accomplished less with work or other activities | | | | 8.86 | 0.003 |
|  | Yes | 69 (34.5) | 20 (55.56) |  |  |
|  | Maybe | 53 (26.5) | 11 (30.56) |  |  |
|  | No | 78 (39) | 5 (13.89) |  |  |
| Worked less carefully | |  |  | 5.67 | 0.02 |
|  | Yes | 55 (27.5) | 17 (47.22) |  |  |
|  | Maybe | 55 (27.5) | 9 (25) |  |  |
|  | No | 90 (45) | 10 (27.78) |  |  |
| How often pain interfered with work | | | | 4.15 | 0.04 |
|  | Little/no interference | 139 (69.5) | 19 (52.78) |  |  |
|  | Moderate interference | 33 (16.5) | 8 (22.22) |  |  |
|  | A lot of interference | 28 (14.0) | 9 (25) |  |  |
| Unable to work due to symptoms in last 4 weeks | | | | 0.75 | 0.39 |
|  | Yes | 79 (39.5) | 17 (47.22) |  |  |
|  | No | 121 (60.5) | 19 (52.78) |  |  |
| Self-reported health rating post-COVID, n (%) | | | | 22.75 | 0.001 |
|  | Poor | 9 (4.5) | 9 (25) |  |  |
|  | Fair | 74 (37) | 20 (55.56) |  |  |
|  | Good | 72 (36) | 5 (13.89) |  |  |
|  | Very Good | 29 (14.5) | 2 (5.56) |  |  |
|  | Excellent | 16 (8) | 0 (0) |  |  |
| Self-reported health rating difference pre/post COVID | | | | 5.88 | 0.02 |
|  | Improved | 2 (1) | 1 (2.78) |  |  |
|  | Same | 100 (50.51) | 9 (25.71) |  |  |
|  | Worse | 98 (49.49) | 26 (74.29) |  |  |

*Kruskal-Wallis test was applied to continuous variables and ordinal variables. Pearson’s chi-squared was applied to categorical variables (categories with less than 5 counts were excluded). For variables with two categories, phi was computed as effect size, while for variables with more than two categories, Cramer’s V was used.

This table provides a summary of baseline characteristics of the 236 participants assigned to the paucisymptomatic (200 patients) and polysymptomatic (36 patients) clusters via the partitioning around medoids clustering method. A p-value threshold of 0.003 was used to denote significant difference between groups. Values are presented as n (%), denoting counts and percentages of patients within each cluster, except for Number of Symptoms and Duration of Symptoms, which are presented as median (IQR).
